# An NMR-Based Approach to Identify Urinary Metabolites Associated with Acute Physical Exercise and Cardiorespiratory Fitness in Healthy Humans—Results of the KarMeN Study

**DOI:** 10.3390/metabo10050212

**Published:** 2020-05-21

**Authors:** Sina Kistner, Manuela J. Rist, Maik Döring, Claudia Dörr, Rainer Neumann, Sascha Härtel, Achim Bub

**Affiliations:** 1 Institute of Sports and Sports Science, Karlsruhe Institute of Technology, 76131 Karlsruhe, Germany; rainer.neumann@ph-karlsruhe.de (R.N.); sascha.haertel@achtzehn99.de (S.H.); achim.bub@kit.edu (A.B.); 2 Department of Physiology and Biochemistry of Nutrition, Max Rubner-Institut, 76131 Karlsruhe, Germany; manuela.rist@mri.bund.de (M.J.R.); maik.doering@mri.bund.de (M.D.); claudia.doerr@mri.bund.de (C.D.)

**Keywords:** metabolomics, urinary metabolome, urinary metabolites, NMR spectroscopy, acute physical exercise, cardiorespiratory fitness, VO_2peak_, exercise metabolomics

## Abstract

Knowledge on metabolites distinguishing the metabolic response to acute physical exercise between fit and less fit individuals could clarify mechanisms and metabolic pathways contributing to the beneficial adaptations to exercise. By analyzing data from the cross-sectional KarMeN (Karlsruhe Metabolomics and Nutrition) study, we characterized the acute effects of a standardized exercise tolerance test on urinary metabolites of 255 healthy women and men. In a second step, we aimed to detect a urinary metabolite pattern associated with the cardiorespiratory fitness (CRF), which was determined by measuring the peak oxygen uptake (VO_2peak_) during incremental exercise. Spot urine samples were collected pre- and post-exercise and 47 urinary metabolites were identified by nuclear magnetic resonance (NMR) spectroscopy. While the univariate analysis of pre-to-post-exercise differences revealed significant alterations in 37 urinary metabolites, principal component analysis (PCA) did not show a clear separation of the pre- and post-exercise urine samples. Moreover, both bivariate correlation and multiple linear regression analyses revealed only weak relationships between the VO_2peak_ and single urinary metabolites or urinary metabolic pattern, when adjusting for covariates like age, sex, menopausal status, and lean body mass (LBM). Taken as a whole, our results show that several urinary metabolites (e.g., lactate, pyruvate, alanine, and acetate) reflect acute exercise-induced alterations in the human metabolism. However, as neither pre- and post-exercise levels nor the fold changes of urinary metabolites substantially accounted for the variation of the covariate-adjusted VO_2peak_, our results furthermore indicate that the urinary metabolites identified in this study do not allow to draw conclusions on the individual’s physical fitness status. Studies investigating the relationship between the human metabolome and functional variables like the CRF should adjust for confounders like age, sex, menopausal status, and LBM.

## 1. Introduction

Over the past decades, a growing body of evidence has demonstrated the inverse relationship between the cardiorespiratory fitness (CRF) and all-cause and disease-specific mortality [1,2]. As one of the most widely investigated physiological parameters [1], the CRF is assessed by measuring the maximal oxygen uptake (VO_2max_) during an incremental exercise test until exhaustion [3]. If the VO_2max_ cannot be determined with certainty, requiring the presence of a plateau in oxygen uptake (VO_2_), the VO_2peak_ as the highest value of VO_2_ attained during an incremental test is frequently used instead [4]. Representing an individual’s ability to take up, transport, and utilize oxygen during sustained physical exercise [5], the CRF is influenced by a variety of factors such as age [6,7], sex [7,8], lean body mass (LBM) [9], or heredity [10]. Furthermore, behavioral factors like diet, smoking, alcohol intake, and particularly physical activity are associated with the CRF [7,11]. Regular physical exercise is known to induce physiological adaptations like an increased cardiac output or skeletal muscle capillarization as well as an augmented activity of mitochondrial enzymes, resulting in a higher aerobic capacity [12]. These alterations are accompanied by a change in skeletal muscle fuel selection during acute exercise, being reflected in an increased ratio of fat to carbohydrate oxidation [13]. However, the molecular mechanisms and metabolic pathways underlying the whole-body and skeletal muscle adaptation to physical exercise still remain to be completely elucidated [14].

The emerging field of metabolomics has the potential to simultaneously analyze a wide range of metabolites [15], thereby facilitating a more comprehensive characterization of exercise-induced changes in human metabolism. As the human metabolome reflects the end-product of interactions between genes, proteins, and the cellular environment [15], investigating the impact of acute or chronic physical activity on a high number and variety of metabolites can provide novel insights into the underlying biochemistry of exercise, possibly hinting at specific metabolic pathways related to the phenotypic response of the human organism to exercise [16,17]. Thus, metabolomics might contribute to the identification of exercise-responsive biomarkers, which are reflective of the individual fitness status. Particularly with regard to the health benefits associated with a high CRF, the investigation of metabolic differences between more and less fit individuals is of great interest not only for sport scientists but also for physicians in the context of exercise prescription and health examination [18].

Until now, several studies have examined both the acute [19,20,21,22,23,24,25,26,27,28,29] and chronic [30,31,32,33,34,35] effects of exercise on the human metabolome by using metabolomics. Although blood represents the most widely used biofluid in metabolomics research [15], recent publications have demonstrated the ability of urine as a non-invasive biological material to reflect acute exercise-induced perturbations in metabolism, e.g., after short-term exercise at maximal intensities [21,22,27] or submaximal endurance exercise [26,29]. However, to the best of our knowledge, no previous study has investigated to what extent the urinary metabolome responds to a standard exercise tolerance test, during which individuals perform a stepwise progressive exercise program until exhaustion. 

A few metabolomics studies have provided first evidence of changes in the blood metabolome due to maximal incremental exercise testing on either bicycle ergometer [19,23,24] or treadmill [19]. Interestingly, Lewis et al. demonstrated that the exercise-induced excursions of some plasma metabolites differentiated between more and less fit individuals, pointing out the possibility that these metabolites could represent important mediators of the salutary effects of exercise and potential biomarkers of physical fitness [19]. With regard to the relationship between acute exercise-related changes in urinary metabolites and the physical fitness status, research is rather scarce. Three studies investigating the alterations in the urinary metabolome due to submaximal endurance exercise [25,26] or short-term intensive exercise [21] either compared the exercise-induced changes in urinary metabolites between groups of different training status [21,26] or aimed to predict the VO_2max_ with the post-exercise urinary metabolite pattern [25]. However, limitations of existing studies are the relatively small sample sizes, ranging from 10 [25] to 22 [21] participants, and the moderate number of quantified analytes (11 [21] or 27 [26] metabolites, respectively) in two of the mentioned studies. Besides, results are partly restricted to young women [21] or middle-aged men [26] and therefore hardly transferable to the general population.

Based on the limited data available on exercise-induced excursions of urinary metabolites being associated with physical fitness and due to the fact that no previous study has investigated to what extent the body’s response to a standardized incremental exercise is reflected in the urinary metabolome, we pursued two major objectives with our study. Firstly, we aimed to characterize acute alterations in urinary metabolites due to a standardized exercise tolerance test on a bicycle ergometer until individual exhaustion, which was performed by 255 healthy women and men of the cross-sectional KarMeN (Karlsruhe Metabolomics and Nutrition) study. The second aim was to investigate if either the urinary metabolites at rest, post-exercise, or the ratio of the post- to pre-exercise metabolite concentrations are associated with the CRF, which was determined by measuring the VO_2peak_. As the metabolomics data were obtained from a comparatively large and heterogeneous population, this study was particularly suitable to analyze in how far urinary metabolite pattern can account for the variation in the VO_2peak_, when simultaneously considering covariates like age, sex, menopausal status, and LBM—all factors determining both the physical fitness [7,36] and the human metabolome [37,38,39,40,41]. A targeted nuclear magnetic resonance (NMR)-based approach was applied to quantify the pre- and post-exercise urinary levels of 47 metabolites. Ranging from organic acids, keto acids, alcohols to purine derivatives and amino acids, the detected metabolites represent a relatively broad spectrum of organic compounds found in the human urinary metabolome [42]. Urine as a biological specimen was chosen because it is easily accessible, stable, and under less homeostatic regulation than other biofluids [42,43].

## 2. Results

### 2.1. Basic Characteristics of Study Participants

Basic characteristics of the KarMeN study participants who were included in the present data analysis are summarized by means and standard deviations in Table 1. Our study sample consisted of 255 healthy individuals, 148 men (58%) and 107 women (42%) with a mean age of 46.1 years. During the incremental exercise test, participants reached a mean absolute VO_2peak_ of 2.83 L min^−1^. As the VO_2peak_ was strongly associated with sex and menopausal status (coefficient of determination, *R*^2^ = 0.59), age (*R*^2^ = 0.35), and LBM (*R*^2^ = 0.69), subsequent correlation and regression analyses were adjusted for these factors (see Section 2.3.1 and Section 2.3.2).

### 2.2. Alterations of Urinary Metabolites in Response to a Standardized Exercise Test

#### 2.2.1. Uni- and Bivariate Analysis

By using an NMR-based approach, a total of 47 urinary metabolites were identified and quantified in spot urine samples collected before and after a standardized incremental exercise test and measured concentrations were normalized to the osmolality of urine samples. To describe and compare the metabolite excursions, fold changes (FCs) between the normalized post- and pre-exercise urinary metabolite concentrations were calculated per participant. In Table 2, alterations in the urinary metabolite levels are summarized, with metabolites being ranked according to their median fold change (FC). The normalized absolute urinary metabolite concentrations before and after the bicycle ergometer test are additionally presented in the Supplementary Table S1 and as boxplots in the Supplementary Figure S1. Boxplots of the FCs of urinary metabolites are provided in the Supplementary Figure S2.

**Figure A1:**
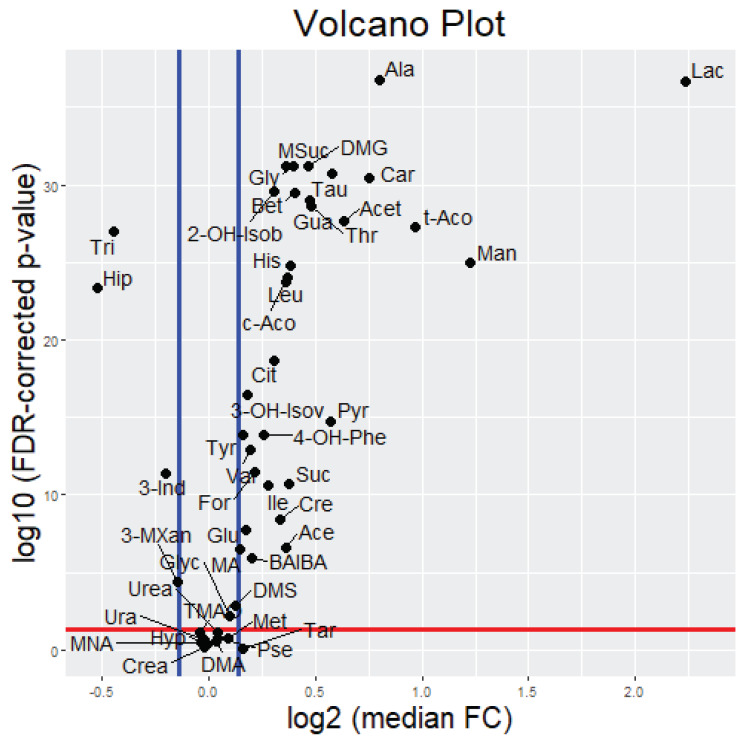
Volcano plot of exercise-related changes in urinary metabolites. Y-axis: represents the statistical significance of metabolite changes (red line: α = 0.05); X-axis: represents the magnitude of metabolite changes (blue lines: FC of 1.1 or $\frac{1}{1.1}$, respectively). Metabolites located in the upper left and upper right part were defined as exercise-responsive metabolites in this study.

Univariate analysis revealed that 37 out of the 47 measured metabolites showed a significantly different urinary excretion post-exercise when compared to pre-exercise. While six of these metabolites (lactate, mannitol, trans-aconitate, alanine, carnitine, and acetate) demonstrated a median FC ≥ 1.5, 17 metabolites showed a median FC between 1.2 and 1.5, 10 metabolites exhibited a median FC between 1.0 and 1.2, two metabolites demonstrated a median FC between 0.8 and 1.0, and two metabolites showed a median FC ≤ 0.8. To facilitate the detection of exercise-responsive metabolites, which were defined as metabolites with FDR-adjusted *p*-values < 0.05 and median FCs ≤ $\frac{1}{1.1}$ or ≥ 1.1, respectively, the significance and magnitude of the post-to-pre-exercise differences are visualized in a volcano plot (see Appendix A Figure A1). Due to the relatively high inter-individual variation observed in the metabolite FC data and due to the known effect of sex on human metabolite profiles, we additionally investigated if there were any sex-related differences in urinary metabolite FCs. However, with the exception of citrate and trans-aconitate, women and men did not show any further significant difference in metabolite FCs. In the Supplementary Table S2, results of the sex-stratified analysis of pre-to-post-exercise changes are presented.

Especially in the exercise-induced excursions of the urinary lactate excretion, a high variance between individuals was detected (see Table 2 and Supplementary Figure S2). When analyzing the associations between the FCs of lactate as the main exercise-responsive metabolite and the FCs of all other metabolites, it was shown that the exercise-related change in the urinary lactate excretion was most closely linked to alterations in the urinary excretion of pyruvate (*r* = 0.76), alanine (*r* = 0.62), methylsuccinate (*r* = 0.57), acetate (*r* = 0.56), and hypoxanthine (*r* = 0.56), see Supplementary Table S3.

#### 2.2.2. Multivariate Analysis 

**Figure 1:**
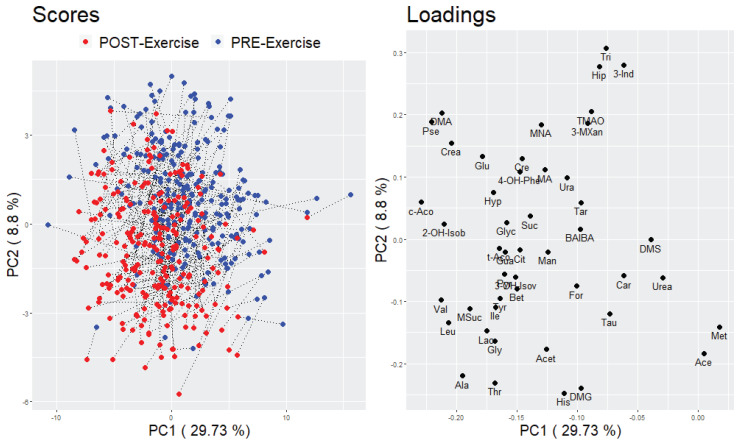
PCA score and loading plot of a combined pre- and post-exercise urinary metabolite data matrix containing 2 × 255 participants and 47 metabolites. The first two principal components are visualized; left: score plot, data points stand for participants and are color coded according to the pre- or post-exercise state; right: loading plot, data points stand for metabolites.

**Figure 2:**
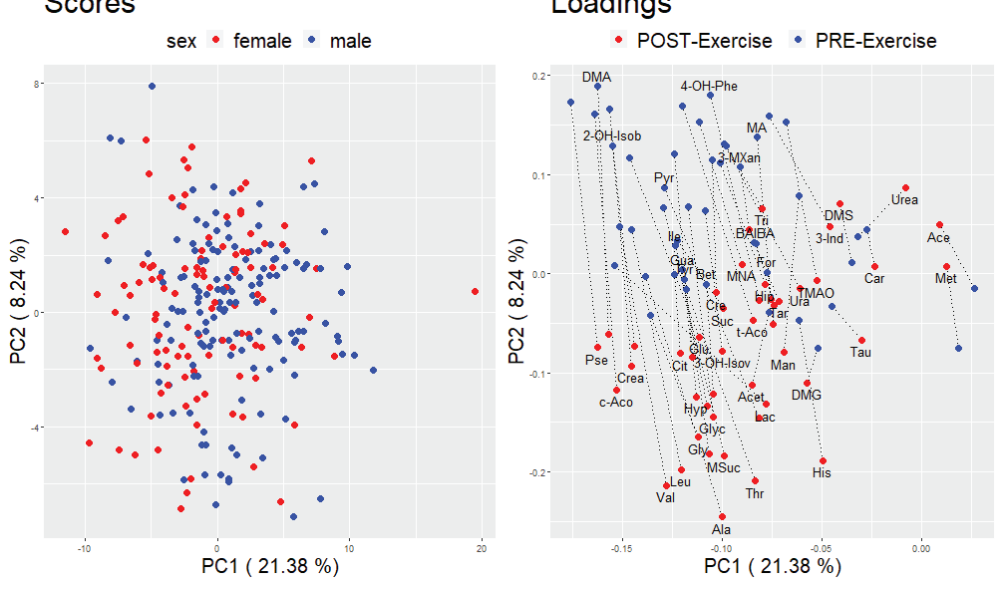
PCA score and loading plot of a combined pre- and post-exercise urinary metabolite data matrix containing 255 participants and 2 × 47 metabolites. The first two principal components are visualized; left: score plot, data points stand for participants and are color coded according to the sex of the participants; right: loading plot, data points stand for metabolites and are color coded according to the pre- or post-exercise state.

Principal component analysis (PCA) was applied as an unsupervised method to describe the differentiation in the metabolite profile between pre- and post-exercise urine samples. Two different approaches were conducted to compare either the participants in the pre- and post-exercise condition (based on a data matrix of 2 × 255 participants and 47 metabolites) or the pre- and post-exercise urinary metabolite profile (based on a data matrix of 2 × 47 metabolites and 255 participants). With regard to the first approach, no clear separation of the participants to either the pre- or post-exercise state was visible in the score plots of the first three principal components (PCs) (see Figure 1 and Supplementary Figure S3a). However, with respect to the second approach, a partial separation between the pre- and post-exercise metabolite profile could be observed in the loading plots of the first three PCs. The metabolite with the greatest change in correlation to the first principle component (PC) between the pre- and post-exercise condition is Lactate (Lac). The separation of the other metabolites was mainly detectable in the second and third PC (see Figure 2 and Supplementary Figure S3b). 

### 2.3. Relationship between the Cardiorespiratory Fitness Status and Urinary Metabolites

Different exploratory approaches were applied to investigate the relationship between urinary metabolite measures and the CRF status. Firstly, we focused on bivariate associations between the VO_2peak_ and single metabolite measures, followed by partial associations independent of phenotypic variables known to determine both the VO_2peak_ and the metabolome (see Section 2.3.1). Secondly, multiple linear regression analyses were conducted to examine the relationship between the VO_2peak_ and all urinary metabolites simultaneously (see Section 2.3.2). To assess which of the metabolite measures (pre-exercise, post-exercise, or the post-to-pre-exercise ratio, i.e., FC) correlates best with the VO_2peak_, all analyses were performed separately for each of the three metabolite parameters. 

#### 2.3.1. Bivariate Analyses

**Figure 3:**
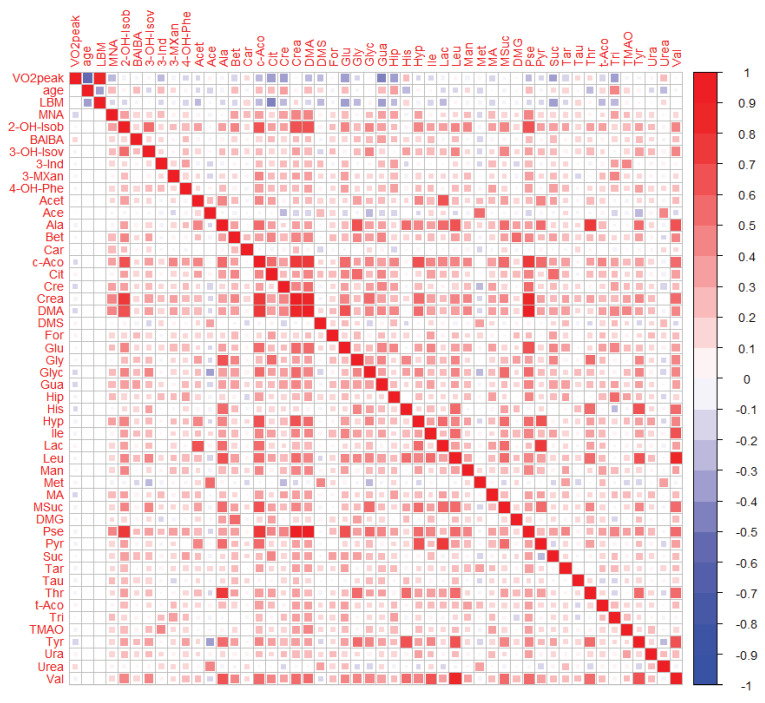
Heat map of correlations between post-exercise urinary metabolites, VO_2peak_, age, and lean body mass (LBM). Right/Up: unadjusted bivariate associations. Left/Low: partial associations adjusted for age, sex, menopausal status, and LBM. Pearson correlations were performed on Van der Waerden-transformed data.

**Figure A2:**
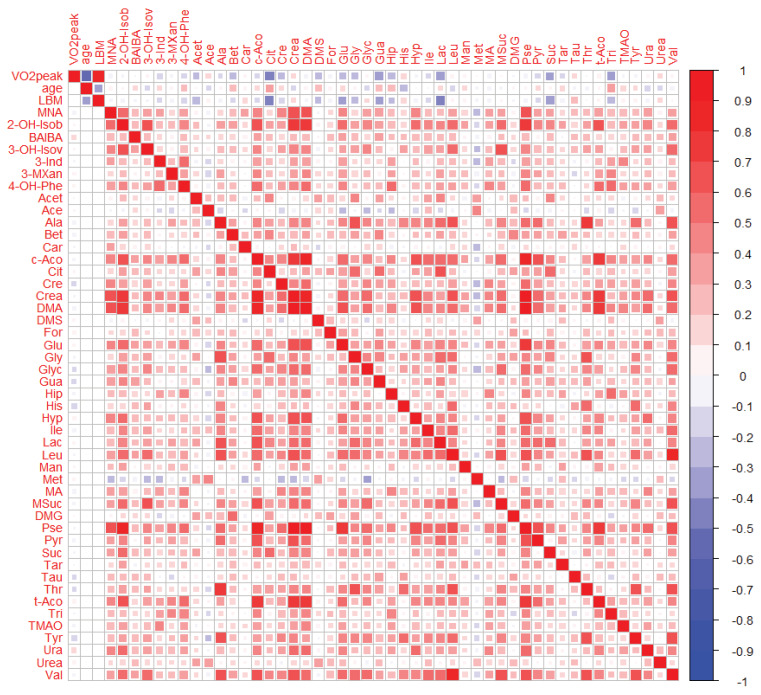
Heat map of correlations between pre-exercise urinary metabolites, VO_2peak_, age, and LBM. Right/Up: Unadjusted bivariate associations. Left/Low: Partial associations adjusted for age, sex, menopausal status, and LBM. Pearson correlations were performed on Van der Waerden-transformed data.

**Figure A3:**
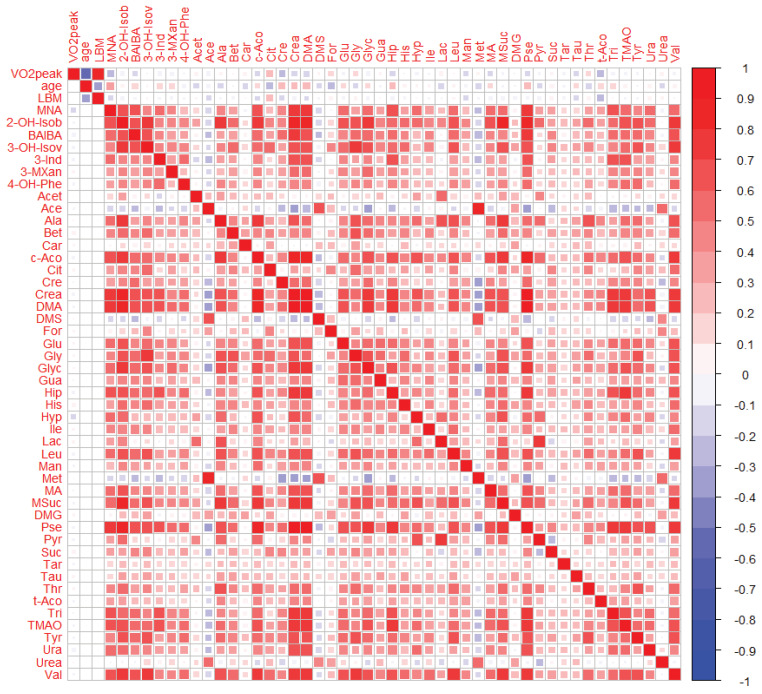
Heat map of correlations between urinary metabolite fold changes, VO_2peak_, age, and LBM. Right/Up: Unadjusted bivariate associations. Left/Low: Partial associations adjusted for age, sex, menopausal status, and LBM. Pearson correlations were performed on Van der Waerden-transformed data.

Pearson correlation coefficients and partial correlations adjusted for age, sex, menopausal status, and LBM were calculated for the associations of the VO_2peak_ with urinary metabolite measures and presented in the Supplementary Table S4. Correlations were considered statistically significant when the 95% confidence intervals did not include zero. A visual comparison of the pairwise associations is additionally provided in correlation heat maps (see Figure 3 for post-exercise measures and Appendix A Figure A2 or Figure A3 for the pre-exercise measures and FCs, respectively).

Results of the unadjusted correlations revealed that 22 pre-exercise urinary metabolites, 24 post-exercise urinary metabolites, and seven FCs of urinary metabolites showed a correlation with the VO_2peak_ which was significantly different from zero. With regard to the pre-exercise metabolite measures, the strongest correlations were observed for citrate (*r* = −0.46), guanidoacetate (*r* = −0.39), lactate (*r* = −0.38), trigonelline (*r* = −0.35), and succinate (*r* = −0.32), see Appendix A Figure A2 (right/up; 1st row). For the post-exercise metabolite measures, guanidoacetate (*r* = −0.41), citrate (*r* = −0.39), gluconate (*r* = −0.36), trigonelline (*r* = −0.36), creatine (*r* = −0.35), hippurate (*r* = −0.34), and succinate (*r* = −0.31) were most strongly associated with the VO_2peak_, see Figure 3 (right/up; 1st row). When regarding the FCs of urinary metabolites, correlations with the VO_2peak_ were comparatively low with *r* ≤ 0.3 or ≥ −0.3, respectively, see Appendix A Figure A3 (right/up; 1st row). 

To examine if the described correlations were dependent on certain influencing factors such as age, sex, menopausal status, and LBM, both the VO_2peak_ and the urinary metabolite measures were adjusted for these factors and correlation analyses were performed on the corresponding residuals. Results of the partial correlation analyses showed that only three pre-exercise urinary metabolites, three post-exercise urinary metabolites and two FCs of urinary metabolites exhibited a partial correlation with the VO_2peak_ which was significantly different from zero. After adjusting for confounding factors, the most correlated urinary metabolite measures included pre-exercise histidine (*r* = −0.17), tyrosine (*r* = −0.16), and uracil (*r* = 0.16), post-exercise tyrosine (*r* = −0.18), 1-methylnicotinamide (*r* = −0.15), and guanidoacetate (*r* = −0.12) as well as the FCs of urinary 1-methylnicotinamide (*r* = −0.13) and hypoxanthine (*r* = −0.13), see Supplementary Table S4 and Figure A2, Figure 3 and Figure A3, respectively (left/down; 1st column). In addition, bivariate correlations of age and LBM with the VO_2peak_ and the urinary metabolite measures confirmed that these factors were strongly correlated with the VO_2peak_ and weakly to moderately associated with some urinary metabolite parameters (see 2nd and 3rd row in Figure 3, Figure A2 and Figure A3, respectively).

#### 2.3.2. Multivariate Analyses

To analyze the relationship between the VO_2peak_ and all urinary metabolites at once, several multiple linear regression procedures were conducted. By adjusting both the VO_2peak_ as the dependent variable and the urinary metabolite measures as the independent variables for age, sex, menopausal status, and LBM, the investigated associations were uncorrelated with these phenotypic variables. 

When all 47 urinary metabolite measures were included into a multiple linear regression model, the adjusted pre-exercise urinary metabolites explained the variation in the adjusted VO_2peak_ to 29.5% (*R*^2^ = 0.295, *R*^2^ (adjusted) = 0.135). While the adjusted post-exercise urinary metabolites similarly accounted for up to 29.6% (*R*^2^ = 0.296, *R*^2^ (adjusted) = 0.137) of the variation in the adjusted VO_2peak_, the inclusion of all 47 adjusted FCs of urinary metabolites resulted in a comparatively lower proportion of explained variance in the adjusted VO_2peak_ (*R*^2^ = 0.141, *R*^2^ (adjusted) = −0.054).

In a next step, we aimed to select the best set of urinary metabolite variables describing the adjusted VO_2peak_. Therefore, two stepwise regression models with either forward or backward selection were performed for each metabolite measure (i.e., pre-exercise, post-exercise and FC). The results of the stepwise regression analyses are presented in the Supplementary Table S5, together with a ranking of the adjusted metabolite variables according to their contribution for explaining the adjusted VO_2peak_. As a criterion for the selection of urinary metabolite variables, the Bayesian information criterion (BIC) was used. For each metabolite measure, the model with the lowest BIC was chosen and the respective metabolite variables were entered into a final multiple linear model. The included urinary metabolite variables as well as criteria for the evaluation and comparison of the three final models are summarized in Table 3.

With regard to the pre-exercise metabolite measures, seven urinary metabolite variables were included into the final model, resulting in an adjusted *R*^2^ of 0.153 and a BIC of 300.9. For the post-exercise metabolite measures, three urinary metabolite variables were selected for the final model, which showed a comparatively lower adjusted *R*^2^ of 0.070 and a higher BIC of 306.6. When regarding the FCs of urinary metabolites, the model with the minimal BIC did not contain any metabolite variables. Thus, the intercept-only model was obtained for the explanation of the adjusted VO_2peak_ based on the urinary metabolite FCs.

Apart from the multiple regression analyses, PCA was performed using either the adjusted pre- or post-exercise urinary metabolite data or the adjusted data on urinary metabolite FCs, in order to detect clusters of participants potentially related to their physical fitness status. The first three principal components were visualized in score and loading plots (see Supplementary Figure S3c–e). In the score plots, the data points were color coded according to the individual VO_2peak_ of the participants. No evident cluster of participants with a similar fitness level could be detected based on the pre- or post-exercise urinary metabolite profile, respectively. Nor did we observe a grouping of participants with a similar fitness level when conducting the PCA on urinary metabolite FCs.

## 3. Discussion

The main finding of this study is that 37 out of 47 measured metabolites showed a significantly different urinary concentration post-exercise when compared to pre-exercise. While the strongest increase was observed for urinary lactate, mannitol, trans-aconitate, alanine, carnitine, and acetate (all demonstrating a median FC > 1.5), the strongest decrease was observed for urinary hippurate and trigonelline (both demonstrating a median FC < 0.8). However, PCA did not permit a clear separation of the pre- and post-exercise urine samples. With regard to the investigated relationship between the VO_2peak_ and urinary metabolites, both bivariate correlation and multiple linear regression analyses revealed only weak associations when adjusting for confounding covariates like age, sex, menopausal status, and LBM. In the following sub-sections, the post-exercise alterations in urinary metabolites (see Section 3.1) as well as their relation to the CRF status of the KarMeN study participants (see Section 3.2) are discussed.

### 3.1. Post-Exercise Alterations in Urinary Metabolites Are Partly Reflective of Energy Metabolism

**Figure A4:**
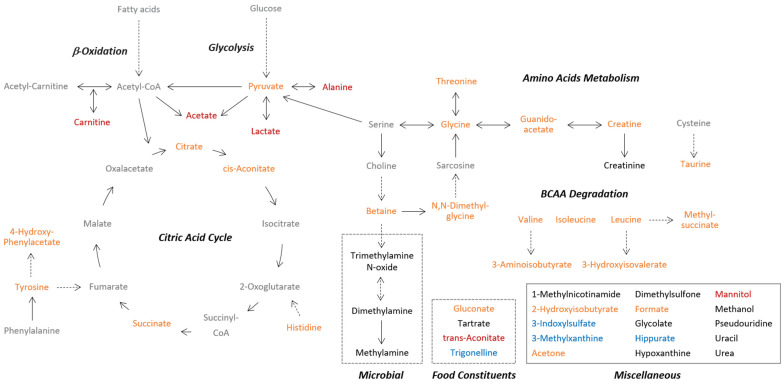
Pathways and origin of identified metabolites. The post-exercise changes of the urinary metabolites are color coded as follows: red, FC ≥ 1.5 and FDR-corrected *p* < 0.05; orange: 1.5 > FC ≥ 1.1 and FDR-corrected *p* < 0.05; black: 1.1 > FC > $\frac{1}{1.1}$ or FDR-corrected *p* ≥ 0.05; blue: FC ≤ $\frac{1}{1.1}$ and FDR-corrected *p* < 0.05; grey: undetected metabolites. BCAA: branched-chain amino acids, FC: fold change between normalized post- and pre-exercise urinary metabolite concentrations, FDR: false discovery rate, *p*: *p*-value obtained by Wilcoxon‘s signed-rank test to analyze significant pre-to-post-exercise differences.

The pre-to-post-exercise comparison of urinary metabolite concentrations confirmed some well-established exercise-induced changes in pathways related to energy metabolism. However, some novel urinary metabolites being altered in response to acute exercise were also revealed. A summary of the origin and pathways of the identified metabolites and the documented effect of acute exercise on urinary metabolite levels is provided in Appendix A Figure A4.

The highest post-exercise increase was observed for urinary lactate (median FC = 4.70), which can be explained by an enhanced carbohydrate catabolism in the exercising muscles. During an incremental exercise test, individuals continuously utilize an increased amount of adenosine triphosphate (ATP). To maintain the muscular ATP resynthesis rate, the required energy is initially provided by mainly aerobic processes and then, with augmented intensity, to an increased degree by the anaerobic energy system [44]. As the end-product of anaerobic glucose breakdown, lactate is released into the blood stream and partly excreted via urine [45]. The documented increase in urinary lactate elimination is in accordance with the results of other exercise metabolomics studies [21,22,25,26,27,29], even though the magnitude of urinary lactate excretion was generally greater in response to maximal exercise protocols [22,27,29]. When compared with other metabolites, we observed a remarkably high inter-individual variation in the post-exercise urinary lactate concentrations. Unfortunately, we were unable to explain this variation by any of the measured physiological parameters and assume it to be a result of the heterogeneous study population and further influencing factors (e.g., the maximally reached exercise intensity, the anaerobic lactic capacity, or the elimination rate of lactate from muscle via blood to urine). However, despite the high variation in the lactate FCs, we noticed that those persons with a high increase in urinary lactate also showed a high increase in urinary pyruvate, alanine and acetate—all metabolites resulting from interconnected metabolic pathways (see Supplementary Table S2 and Appendix A Figure A4). For instance, the post-exercise increase of urinary pyruvate (median FC = 1.48) can equally be seen as a result of an increased glycolysis. When the pyruvate production rate during exercise is higher than the capacity of the mitochondria to take up pyruvate, the skeletal muscle has to remove it from the cytosol [46]. Thus, pyruvate is either directly released into the blood or previously converted to lactate via lactate dehydrogenase reaction [46] or to alanine via transamination [45], thereby providing substrates for gluconeogenesis in the liver [45,47]. Our results demonstrated a median 1.74-fold increase in the urinary alanine excretion due to exercise, which is in line with previous work [21,26,27,29]. As the alanine flux into the bloodstream during exercise was shown to be higher than the alanine uptake by the liver [48], it can be assumed that this amino acid accumulated in blood and was therefore excreted via urine after the incremental test. Next to pyruvate and alanine, the post-exercise increase in urinary lactate was accompanied by a median 1.55-fold increase in urinary acetate, confirming the results of previous studies investigating the effect of acute exercise on the urinary metabolome [21,26,27]. Two mechanisms for explaining this observation are discussed in the literature. Firstly, the rise in urinary acetate is likely to be the consequence of an increased hydrolysis of acetyl-CoA, which was produced from pyruvate but could not entirely enter into the tricarboxylic acid (TCA) cycle and was therefore released into the blood [27,49]. Secondly, acetate might be directly produced in a radical-removing reaction from pyruvate, which seems able to convert reactive oxygen species into carbon dioxide and acetate [50,51].

With regard to the remaining amino acids (Gly, His, Ile, Leu, Tau, Thr, Tyr, Val), we observed a post-exercise increase in their urinary excretion, reaching from a median 1.12-fold (Tyr) to a median 1.49-fold (Tau) rise. This observation points to higher circulating amino acid concentrations, but it remains speculative if this was due to an increased exercise-induced protein degradation or to a higher amino acids availability owing to the breakfast participants consumed after the first spot urine collection. Although the rise in urinary alanine was consistent across several exercise-related metabolomics studies, this global increase in amino acids has not been detected in previous work. While Siopi et al. could confirm a higher urinary excretion of glycine, histidine, taurine, tyrosine, and threonine 2 h after high-intensity interval and continuous moderate exercise [29], other studies documented an unchanged urinary excretion of taurine [22,27], isoleucine, leucine, and valine [22] or a decreased level in urinary glycine [22,26,27], leucine [25], histidine [22], threonine [25], and tyrosine [27], respectively, in response to the different exercise protocols. With respect to the increase in urinary taurine, a semi-essential amino acid highly concentrated in human muscles [52], it can be supposed that the acute exercise resulted in an increased muscular taurine release. Consistent with this explanation, previous work has demonstrated an exercise-induced release of taurine from contracting muscles due to osmoregulatory processes, leading to a higher taurine level in both blood and urine [53]. However, as the urinary excretion of taurine furthermore depends on the dietary taurine intake [54], it cannot be completely excluded that the intake of taurine-containing food like cheese at breakfast led to the observed increase in urinary taurine excretion.

Four further metabolites related to amino acid metabolism showed a post-exercise increase in their urinary level, namely methylsuccinate (median FC = 1.28) and 3-hydroxyisovalerate (median FC = 1.13), both byproducts of the leucine degradation pathway [55,56], 4-hydroxyphenylacetate (median FC = 1.19), a degradation product of tyrosine, and 3-aminoisobutyrate (median FC = 1.15), a valine degradation product and recently discovered myokine [57]. The amino acid derivative N,N-dimethylglycine (median FC = 1.38) and guanidoacetate (median FC = 1.39), an intermediate in the metabolic pathway of several amino acids, were also reported to be elevated in the post-exercise urine samples. To our knowledge, no earlier metabolomics studies have reported exercise-related changes in the urinary concentration of these metabolites.

With regard to metabolites of the TCA cycle (i.e., citrate, cis-aconitate and succinate), a median 1.24- to 1.30-fold increase in their urinary levels was observed post-exercise. A significant rise in urinary succinate has previously been documented in response to a 30 s maximal sprint [21], whereas a decrease in both urinary succinate [26,27] and citrate [22,26,27,29] was reported after intermittent [22,27,29] or submaximal exercise [26,29]. Compared to this, other studies showed that acute exercise was followed by an increase in plasma TCA cycle intermediates, suggesting that these compounds spilled over from muscle into the circulation due to an increased TCA cycle flux [58,59]. However, whether the increased post-exercise urinary excretion of TCA cycle metabolites in our study also can be traced back to this explanation, remains unclear. Interestingly, we observed that women had a lower fold change of urinary citrate than men (median 1.16- vs. 1.29-FC). This might hint at sex-specific differences in exercise-related citrate turnover.

To our knowledge, this is the first study reporting a post-exercise increase in the urinary excretion of carnitine (median FC = 1.68). Carnitine, which can either be synthesized from the amino acid lysine or ingested through diet, is largely stored in the skeletal muscle, where it is involved in the translocation of long-chain fatty acids into the mitochondrial matrix for subsequent β-oxidation and the buffering of accumulating acetyl-CoA [60]. Previous work has shown that acute exercise results in a higher muscle and plasma concentration of acetylcarnitines (reflecting an enhanced pyruvate and fatty acids oxidation) or long-chain acylcarnitines (reflecting an increased mobilization of free fatty acids) [24,61,62,63,64], accompanied by a decrease in muscular [62,65] and blood [61,62] free carnitine. However, neither a decreased nor an increased urinary excretion of free carnitine has been noticed so far [22,27,65]. 

In addition to carnitine, urinary mannitol and trans-aconitate demonstrated a more than 1.9-fold increase in response to the incremental exercise test. Mannitol is a polyol found in many foods and used as an artificial sweetener. It is produced by microorganisms [66] and cannot be metabolized by humans [67]. Although an exercise-related increase of urinary mannitol was also shown by Siopi et al. [29], the reasons for this alteration are currently unclear. As mannitol in urine seems to show diurnal variation [68], an effect of time on the mannitol excretion cannot be excluded. Trans-aconitate is an organic acid present in plants like wheat or soybean and seems not to be degraded in human metabolism [69]. Since participants of this study consumed wheat bread before exercising, it is presumable that the increase in urinary trans-aconitate was due to its dietary intake and not owing to acute physical exercise. That women demonstrated a higher increase in urinary trans-aconitate than men (median 2.33- vs. 1.53-FC) could indicate sex-related differences in the biokinetics of absorbed trans-aconitate.

With regard to betaine (median FC = 1.32), an increased urinary excretion has previously been documented in response to intermittent and submaximal endurance exercise [29]. Betaine functions as an organic osmolyte and methyl donor; it is endogenously produced or ingested through foods like wheat or spinach [70]. However, as the urinary betaine excretion seems to be relatively stable, not being considerably affected by food intake or hydration status [71], we are currently unable to explain the observed increase in urinary betaine. Similarly, it is unclear why the level of urinary acetone (median FC = 1.28) increased after exercise. The ketone body is produced in the liver when there is a shortage of carbohydrates, e.g., during prolonged exercise or in the fasting state [72]. Concerning creatine, there is one study confirming the post-exercise increase in its urinary excretion [29] and one study showing opposite results [25]. Altogether, the observed increases in urinary creatine, formate, gluconate, and methylamine can hardly be explained and might be due to uncontrolled variation. However, one interesting finding is the post-exercise rise in the urinary excretion of 2-hydroxyisobutyrate (median FC = 1.24), which was already shown in previous exercise metabolomics studies [27,29]. Urinary 2-hydroxyisobutyrate is mainly known as a degradation product of gasoline additives entering the body through inhalational exposure [73]. Recently, it was also suggested to be a gut microbial metabolite [74] and an indicator of the increased lactate production due to alcohol consumption [75]. As the same carrier protein seems to be responsible for the (re)absorption of both lactate and 2-hydroxyisobutyrate in the kidney, it was assumed that elevated lactate levels inhibit the 2-hydroxyisobutyrate reabsorption rate, thus contributing to an increase in its urinary excretion [75]. However, it remains largely speculative if this mechanism also underlay the exercise-related increase of urinary 2-hydroxyisobutyrate in this study.

The four metabolites showing a decrease in the post-exercise urine samples were identified as either microbial cometabolites (3-indoxylsulfate [76] and hippurate [77]) or diet-related metabolites (3-methylxanthine [78] and trigonelline [79]; both markers of coffee consumption). As we could not find a relevant link between these metabolites and acute exercise, we assume that the observed decline in their urinary levels was due to uncontrolled variation.

Rather unexpectedly, no change was documented in the urinary excretion of hypoxanthine, the final ATP degradation product, which is known to be released during exercise from muscles to blood and excreted via urine [80]. In agreement with its proposed function as an indicator of exercise-related energetic stress [80,81], hypoxanthine was increased in the post-exercise urine samples of several metabolomics studies [21,22,25,26,27,29]. The discrepant results could be explained by a comparatively lower exercise intensity or duration in the KarMeN study or a time point of sample taking that was too early to capture the post-exercise rise of hypoxanthine in urine. 

With regard to our multivariate approaches, we could not observe a clear distinction between the pre- and post-exercise urine samples (see Figure 1), suggesting that the incremental exercise test did not cause a substantial variation in the overall urinary metabolite profile. Conversely, the PCA loading plots in Figure 2 and Figure S3b showed a partial separation between the pre- and post-exercise urinary metabolites which was predominantly detectable in the second and third PC. These results indicate that there was a variation in the urinary metabolite profile which, however, could not primarily and solely be attributed to the performed exercise. Although the results of our univariate analysis revealed that 37 out of 47 urinary metabolites significantly changed in response to exercise, it was also apparent that the degree of changes in urinary metabolites was less profound than in other exercise-related metabolomics studies [21,22,27,29]. Furthermore, we generally observed a high inter-individual variability in urinary metabolite concentrations and calculated FCs, which was probably due to the heterogeneous study population and the fact that the dietary intake on the day before the bicycle ergometry was not standardized. Taken together, discrepancies regarding the type, duration, and intensity of exercise as well as the time point of sample taking and the selected study population only allowed a limited comparison of our results to other studies. What additionally has to be taken into account when interpreting our findings is that large sample sizes like in this study can amplify the detection of statistically significant metabolite changes, which are, however, not necessarily biologically relevant [82]. However, as we could detect known exercise-related metabolite changes being reflected in the urinary metabolome, we conclude that metabolomics studies focusing on urine need to control for physical activity.

### 3.2. Urinary Metabolites at Rest and after Exercise Are Not Substantially Related to Physical Fitness

Despite a high inter-individual variance in both the analyzed urinary metabolites and the VO_2peak_, we revealed only weak to moderate associations between the CRF and the pre- and post-exercise urinary metabolite profile or the exercise-related metabolite FCs, respectively. When comparing the results of the single and partial correlation analyses, it became obvious that the associations between the VO_2peak_ and the urinary metabolite variables were strongly influenced by age, sex, menopausal status, and LBM. After adjusting for these covariates, only few single urinary metabolites showed weak correlations with the VO_2peak_ which were significantly different from zero, namely pre-exercise urinary histidine (*r* = −0.17), tyrosine (*r* = −0.16), and uracil (*r* = 0.16), post-exercise urinary 1-methylnicotinamide (*r* = −0.15), guanidoacetate (*r* = −0.12), and tyrosine (*r* = −0.18) and fold changes of urinary 1-methylnicotinamide (*r* = −0.13) and hypoxanthine (*r* = −0.13). Even if no reliable urinary marker for the VO_2peak_ could be detected in this study, our results hint at a few potentially interesting urinary metabolites that are independently of known covariates linked to the VO_2peak_. For example, the slight negative association between pre-exercise urinary histidine and the VO_2peak_ is in line with a previous study documenting a significant lower urinary histidine excretion in a high-fitness group compared to a low-fitness group [31]. As opposed to this, the weak negative association between the FC of urinary hypoxanthine and the VO_2peak_ does not confirm previous results from Mukherjee et al. who showed that more fit individuals demonstrated a higher increase in urinary hypoxanthine after a submaximal endurance exercise than less fit participants [26]. The discrepant results could be explained by distinct approaches to detect differences in post-exercise metabolite alterations or by different study populations. We used correlation and regression analyses instead of group comparisons to be able to draw conclusions across a broad range of fitness levels in healthy women and men. Previous studies characterizing the human urinary metabolome in relation to acute exercise and the fitness status mainly observed fitness-associated differences in exercise-induced metabolite changes between pre-defined groups of either athletes or untrained persons [21,26]. We suppose that maybe an athletic background with several years of training and possibly a distinct genetic background is necessary to substantially alter metabolic pathways. 

Our multivariate approaches, which analyzed the relationship between the adjusted VO_2peak_ and all urinary metabolites at once, showed that there was no evident metabolic pattern related to the fitness status in neither the pre- nor the post-exercise condition. Thus, it can be summarized that the 47 analyzed metabolites do not strongly account for the variation in the CRF after having adjusted for age, sex, menopausal status, and LBM. When furthermore comparing the ability of selected urinary metabolite variables to explain the variation in the adjusted VO_2peak_, we noticed that the adjusted pre-exercise urinary metabolites resulted in a comparatively better model in the sense of BIC than the adjusted post-exercise urinary metabolites or respective FCs. This observation indicates that acute changes in urinary metabolites might primarily be driven by the energy requirements due to the performed exercise and less reflective of the individual’s fitness status.

### 3.3. Strengths and Limitations

The present study has several strengths and limitations. Firstly, a high number of healthy women and men with a wide age range were included in this study, which was characterized by highly standardized clinical and physiological examinations and a strictly scheduled experimental setting. Thus, we were able to investigate how urinary metabolites are associated with both acute physical exercise and the CRF status in a comparatively large population. Additionally, owing to the comprehensive characterization of study participants, potential confounders like age, sex, LBM, and menopausal status could be considered. To furthermore minimize the variability in metabolomics measurements, pre-menopausal women were scheduled for the bicycle ergometry within the luteal phase of their menstrual cycle. One limitation of this study is that the time point of post-exercise urine sampling was not strictly controlled. Participants were told to collect their first available spot urine after having completed the incremental test, which was approximately 15–30 min post-exercise. Besides, as the participants collected the pre-exercise spot urine samples before breakfast, it has to be taken into account that the urinary metabolite changes could be related to both the post-exercise and the post-prandial state. Additionally, no causal relationships between the individual’s fitness status and the metabolome can be proven due to the cross-sectional study design. 

Even though our NMR-based analysis had the advantage to allow the absolute quantification of urinary metabolites with known identity and of different chemical classes, this targeted approach was limited to a comparatively small selection of urinary compounds. In future studies, it would be appropriate to analyze the acute and chronic effects of physical exercise on a broader spectrum of metabolites in both blood and urine.

## 4. Materials and Methods 

### 4.1. Subjects and Study Design

The Karlsruhe Metabolomics and Nutrition (KarMeN) study is a cross-sectional study which was performed between March 2012 and July 2013 at the Division of Human Studies of the Max Rubner-Institut in Karlsruhe, Germany. The main objective of the KarMeN study was to investigate the impact of sex, age, body composition, and major lifestyle factors like diet and physical activity on the metabolome of healthy women and men. Detailed information about inclusion and exclusion criteria as well as a detailed description of the study design were provided in Bub et al. [83]. Briefly, 301 healthy, non-smoking individuals (172 men, 129 women) between 18 and 80 years were included. Volunteers were thoroughly characterized by anthropometric and clinical examinations. The body composition was assessed by dual-energy X-ray absorptiometry (DXA; Lunar iDXA, GE Healthcare, Germany) and lean body mass (LBM) and fat mass were calculated with the supplementary software enCOREv16. Blood hemoglobin was measured by a certified clinical chemistry laboratory (MZV Labor PD Dr. Volkmann, Karlsruhe, Germany). Furthermore, resting energy expenditure and CRF were assessed and data on regular physical activity and menopausal status in women were collected. As the menstrual cycle is known to influence metabolite profiles [84], all premenopausal women were scheduled for examinations within their luteal phase. The study was approved by the ethics committee of the State Medical Chamber of Baden-Württemberg, Stuttgart, Germany and was conducted in accordance with the declaration of Helsinki. The study was registered at the German Clinical Trials Register (DRKS00004890). The WHO universal trial number is U1111-1141-7051. Written informed consent was obtained from all participants prior to entering the study.

### 4.2. Exercise Examination Day and Urine Sample Collection

On the exercise examination day, participants entered the study center early in the morning. Before the bicycle ergometry started, volunteers passed a resting phase for the indirect calorimetry measurement (40 min), consumed a standardized breakfast consisting of two slices of wheat bread with butter and either cheese, ham, or jam (20 min) and had a second resting phase for answering the International Physical Activity Questionnaire (20 min). Immediately after entering the study center, all participants provided fasting spot urine into 100 mL polypropylene collection cups (Sarstedt, Nümbrecht, Germany). This was approximately 90 min before the bicycle ergometry started. In addition to that, the first available spot urine after the completion of the exercise test was obtained from all participants approximately 15–30 min post-exercise.

Participants performed a standardized exercise test on a bicycle ergometer (Ergobike medical, Daum, Fürth, Germany) until individual maximal performance was reached, see Biniaminov et al. [85]. Briefly, according to the WHO-loading protocol [86], each participant started pedaling at 25 Watt and workload was then augmented by 25 Watt every 2 min until individual exhaustion. Respiratory gas exchange was measured breath-by-breath by using a cardiopulmonary exercise testing system (MetaMax 3B, Cortex, Leipzig, Germany). As a measure of physical fitness, the peak oxygen uptake (VO_2peak_) was determined. The VO_2peak_ was defined as the highest achieved oxygen uptake during the test. It is either expressed as an absolute value in L min^−1^ or relative to the body weight in mL kg^−1^ min^−1^. Further important endpoints of the incremental test were the maximal power (P_max_), maximum heart rate (HR_max_), and the power at individual anaerobic threshold (P_IAT_). The P_IAT_ was determined by the Ergonizer software (Ergonizer, Version 4, Freiburg, Germany) after having measured lactate in capillary blood samples taken from the individual’s earlobe before, during and after the test. Throughout the entire procedure, the heart rate was recorded by a heart rate monitor (T31 coded, Polar Electro GmbH Deutschland, Büttelborn, Germany). Additionally, a continuous hemodynamic monitoring was conducted by running a 12-channel electrocardiogram (CardioDirect 12, DelMar Reynolds GmbH, Feucht, Germany) and by measuring the blood pressure every 2–3 min on the right upper arm (boso Carat professional, Bosch + Sohn, Jungingen, Germany). Break-off criteria for the maximal exercise test were predefined according to national ergometry standards and listed in the following: ST-segment depression > 3 mm, ST-segment elevation > 1 mm, acute hypertension with systolic blood pressure > 230 mmHg or diastolic blood pressure > 115 mmHg, appearance of angina pectoris symptoms or severe dyspnea.

### 4.3. Urine Sample Preparation

After urine sample collections, all urine specimens were centrifuged at 1850 g at 4 °C for 10 min and transferred into prechilled cryovials. They were initially frozen at −20 °C for one day and then cryopreserved at −196 °C until analysis. As previously shown, this procedure does not affect metabolomics results [87].

### 4.4. 1H-NMR Analysis

In order to identify and quantify a relatively broad range of urinary metabolites before and after the cycle ergometry, all urine samples were analyzed by 1D-^1^H-NMR spectroscopy. As previously described [37], urine samples were centrifuged and supernatants were mixed with 10% of a buffer containing 1.5 M KH_2_PO_4_, 2 mM NaN_3_, and 5.8 mM trimethylsilylpropanoic acid (TSP) in D_2_O at pH 7.4 in 5 mm NMR tubes (Duran, purchased from Roth GmbH & Co KG, Karlsruhe, Germany). Samples were measured at 300 K on a Bruker 600 MHz spectrometer (either AVANCE III equipped with a ^1^H,^13^C,^15^N-TCI inversely detected cryoprobe or AVANCE II with 1H-BBI room temperature probe (Bruker BioSpin GmbH, Rheinstetten, Germany)) equipped with either SampleXpress or BACS sample changer using a 1D nuclear overhauser enhancement spectroscopy (NOESY) experiment with presaturation for water suppression. A prescan delay of 4 s was used together with a mixing time of 10 ms. Pulse lengths were determined automatically by the Bruker AU program, pulsecal. 64 k complex data points corresponding to a sweep width of 20 ppm were recorded. All spectra were treated identically using an exponential apodization function, introducing an additional linewidth of 0.3 Hz and automated phasing, baseline correction, and referencing using the Bruker macro, apk0.noe. Quality control (QC) samples were prepared by pooling urine samples from all participants. On each measurement day, at least three QC samples were analyzed along with approximately 70 urine study samples, ensuring the comparability of the spectra over the time. Since alignment of metabolites in urine spectra for non-targeted analysis is still very challenging, a targeted NMR-based metabolomic approach was applied. The identification and quantification of 47 urinary metabolites, including organic acids, amino acids, amines, sugars, sugar alcohols, and others was carried out with the Chenomx NMR Suite 8.4 (Chenomx, Edmonton, AB, Canada). Further metabolites could either not be detected in each urine sample or not identified and/or quantified with sufficient confidence. Imprecision was generally <15%, with few exceptions. No systematic variation between the two spectrometers was observed. Metabolite concentrations were normalized to osmolality, thus controlling for variations in urine dilution [88], and results are given in µmol/L per mOsm/kg urine. The osmolality of urine samples was determined by freezing point depression using an Advanced Instrument Micro-Osmometer model 3MO (Norwood, MA, USA). The normalized metabolite data as well as the technical precision of the NMR analysis are presented in the Supplementary Table S1.

### 4.5. Data Handling and Statistical Analysis

We excluded 46 individuals due to missing spirometry data (*n* = 40), outlying urinary metabolite concentrations (*n* = 5) and implausible data on resting heart rate (*n* = 1). Thus, data from 255 individuals were included into the following analyses. Descriptive characteristics of the study participants are presented as mean and standard deviation (SD) for the total study sample and separately for women and men. In the Supplementary Table S1, the median and percentiles (25th, 75th) of basic characteristics are additionally provided. Sex differences in basic characteristics were examined by Welch’s *t*-test.

For each of the 47 metabolites, fold changes (FCs) between the normalized post- and pre-exercise concentrations were calculated per participant and shown as median and percentiles (25th, 75th). Compared to absolute differences in metabolite concentrations, FCs are unitless and therefore easier to interpret, allowing the direct comparison of exercise-induced excursions between different metabolites. In the Supplementary Table S1, the entire data on absolute metabolite concentrations and FCs of urinary metabolites are additionally summarized by the arithmetic mean and SD. As the urinary metabolite concentrations did not follow a normal distribution, the data were subjected to nonparametric univariate statistical analysis. In order to identify urinary metabolites that were significantly different between pre- and post-exercise, Wilcoxon’s signed-rank tests were used. The false discovery rate (FDR) was applied to correct the obtained *p*-values for multiple hypothesis testing, using the method of Benjamini and Hochberg with an extension to the corresponding simultaneous tests. Adjusted *p*-values were compared to the level of statistical significance, which was set at α = 0.05. Metabolites with FDR-corrected *p*-values < 0.05 and median FCs ≤ $\frac{1}{1.1}$ or ≥ 1.1 were considered as exercise-responsive metabolites. A volcano plot was provided to visualize the magnitude (FCs) and significance (FDR-corrected *p*-values) of differences in urinary metabolites. To compare the metabolite FCs between women and men, the Wilcoxon rank-sum test was used and FDR-corrected *p*-values were reported (see Supplementary Table S2). 

Due to the non-normal data distribution and the occurrence of outliers in the urinary metabolite data, all variables were transformed into Van der Waerden scores prior to the following analyses. In detail, by using this rank based inverse normal transformation, the data were converted into ranks, transformed to a scale between 0 and 1 and, then, the corresponding standard normal quantiles were calculated. To investigate the relationships between the FCs of urinary metabolites, Pearson correlation coefficients (*r*) with 95% confidence intervals (CIs) were calculated. Furthermore, PCA analysis was conducted on the transformed metabolite data, which were centered and scaled to unit variance, in order to visualize the main variability on a reduced dimensionality. Two different approaches were applied. The first approach was based on a data matrix of 2 × 255 participants from the pre- and post-exercise condition and 47 metabolites. The second approach dealt with a data matrix of 255 participants and 2 × 47 metabolites from the pre- and post-exercise condition. Relationships between the VO_2peak_ and either pre- and post-exercise urinary metabolite concentrations or urinary metabolite FCs were analyzed by Pearson correlation. To remove any confounding association, we also performed partial correlation analyses by using the residuals from linear regressions, where both the transformed VO_2peak_ and the transformed metabolite variables were regressed on age, sex, menopausal status, and LBM.

In a next step, multiple linear regression procedures were conducted to analyze the relationship between the VO_2peak_ and all urinary metabolite variables simultaneously. Three different models were calculated with the previously obtained residuals of VO_2peak_ as the dependent variable and the residuals of (a) pre-exercise urinary metabolites, (b) post-exercise urinary metabolites, and (c) urinary metabolite FCs, respectively, entering as independent variables. By this construction, the analyzed relationships were confounder-adjusted and hence uncorrelated with known determinants of both the VO_2peak_ and the urinary metabolome. In more detail, two stepwise regression models with either forward or backward elimination were performed for each (a), (b), and (c) to obtain a ranking of the adjusted metabolite variables according to their contribution for explaining the adjusted VO_2peak_. For the selection and elimination processes, the Bayesian information criterion (BIC) was used. Finally, based on the results of the stepwise regression procedures, the models with the minimum BIC were chosen in order to select those metabolite variables which should enter into a suitable final model. The adjusted coefficients of determination (*R*^2^ (adjusted)) were used to compare the three obtained final models with respect to their potential in explaining the variation of the adjusted VO_2peak_. Statistical analysis was performed using SAS JMP 11.0.0. (SAS Institute Inc. 2013, Cary, NC, USA) and the software R Version 3.6.0 [89], using the packages XLConnect [90], ggplot2 [91], ggpubr [92], corrplot [93], and ggrepel [94]. The R-script for figure generation is provided in the Supplementary File S1.

## 5. Conclusions

We investigated the effect of a standardized exercise tolerance test on 47 urinary metabolites of 255 healthy women and men. Besides, we analyzed whether the VO_2peak_ measured in the incremental test is associated with exercise-related metabolite excursions and pre- or post-exercise urinary metabolite pattern. Although PCA analysis did not show a clear separation of the pre- and post-exercise urine samples, univariate analysis revealed significant pre-to-post-exercise alterations in the urinary excretion of 37 metabolites—with the strongest increase being observed for lactate, mannitol, trans-aconitate, alanine, carnitine, and acetate. However, only weak relationships between the VO_2peak_ and single urinary metabolites or urinary metabolite pattern, respectively, could be revealed after adjusting for covariates like age, sex, menopausal status, and LBM. Our findings indicate that the analyzed urinary metabolites partly reflect acute exercise-related changes in the human metabolism, but do not allow to conclude about the individual’s fitness status. We recommend future urinary metabolomics studies to control for acute physical exercise and to consider the mentioned confounders if investigating functional variables like the VO_2peak_.

## Figures and Tables

**Table 1:** Basic characteristics of KarMeN (Karlsruhe Metabolomics and Nutrition) study participants (total and stratified by sex).

Characteristics of Participants	Total (*n* = 255)	Men (*n* = 148)	Women (*n* = 107) ^1^
Age (years)	46.1 ± 16.9 *	42.8 ± 17.6	50.7 ± 14.7
Body weight (kg)	72.5 ± 11.6 *	78.5 ± 10.0	64.2 ± 8.1
Height (cm)	174.6 ± 9.6 *	180.1 ± 7.5	166.9 ± 6.5
BMI (kg (m²)^−1^)	23.7 ± 2.8 *	24.2 ± 2.7	23.1 ± 2.9
LBM (kg)	50.6 ± 10.4 *	58.0 ± 6.6	40.3 ± 3.8
Fat mass (%)	27.9 ± 8.7 *	23.2 ± 6.5	34.5 ± 6.7
Hemoglobin (g dL^−1^) ^2^	14.4 ± 1.1 *	15.0 ± 0.9	13.5 ± 0.8
BP systolic (mmHg)	123.6 ± 15.4 *	127.2 ± 13.6	118.6 ± 16.4
BP diasystolic (mmHg)	83.1 ± 10.5	83.9 ± 10.2	82.1 ± 10.9
HR_rest_ (bpm)	62.4 ± 9.4 *	59.9 ± 9.3	65.8 ± 8.6
VO_2peak_, absolute (L min^−1^)	2.83 ± 1.00 *	3.46 ± 0.81	1.96 ± 0.42
VO_2peak_, relative (mL kg^−1^ min^−1^)	38.8 ± 11.6 *	44.5 ± 10.7	30.9 ± 7.4
P_IAT_ (watt)	144.5 ± 46.2 *	170.5 ± 41.1	108.4 ± 22.6
P_max_ (watt)	215.2 ± 69.1 *	257.0 ± 56.0	157.5 ± 36.0
HR_max_ (bpm)	170.8 ± 16.8 *	174.1 ± 16.1	166.2 ± 16.8

All values in mean ± SD; ^1^ *n* = 49 in the pre- and *n* = 58 in the post-menopausal state on the examination day; ^2^ *n* = 254 (total), *n* = 106 (women); * *p* < 0.05, significant difference between women and men by Welch’s *t*-test; BP: blood pressure; BMI: body mass index; HR_max_: maximum heart rate; HR_res_: resting heart rate; LBM: lean body mass; P_IAT_: power at individual anaerobic threshold; P_max_: maximal power; VO_2peak_: peak oxygen uptake.

**Table 2:** Changes in urinary metabolites after a standardized incremental exercise test.

Nr.	Metabolite (Abbreviation)	Median Fold Change (25th, 75th Percentiles)		FDR-Corrected *p*-Value
**1**	*Lactate (Lac)*	4.70	(1.64, 32.98)	<0.0001
2	*Mannitol (Man)*	2.34	(1.30, 4.29)	<0.0001
3	*trans-Aconitate (t-Aco)*	1.96	(1.05, 3.49)	<0.0001
4	*Alanine (Ala)*	1.74	(1.38, 2.20)	<0.0001
5	*Carnitine (Car)*	1.68	(1.24, 2.37)	<0.0001
6	*Acetate (Acet)*	1.55	(1.14, 2.68)	<0.0001
7	*Taurine (Tau)*	1.49	(1.19, 2.02)	<0.0001
8	*Pyruvate (Pyr)*	1.48	(0.88, 3.57)	<0.0001
9	*Threonine (Thr)*	1.40	(1.12, 1.86)	<0.0001
10	*Guanidoacetate (Gua)*	1.39	(1.14, 1.71)	<0.0001
11	*N,N-Dimethylglycine (DMG)*	1.38	(1.13, 1.68)	<0.0001
12	*Betaine (Bet)*	1.32	(1.12, 1.58)	<0.0001
13	*Glycine (Gly)*	1.32	(1.13, 1.63)	<0.0001
14	*Histidine (His)*	1.30	(1.07, 1.62)	<0.0001
15	*Succinate (Suc)*	1.30	(0.92, 1.76)	<0.0001
16	*cis-Aconitate (c-Aco)*	1.29	(1.08, 1.74)	<0.0001
17	*Methylsuccinate (MSuc)*	1.28	(1.12, 1.54)	<0.0001
18	*Leucine (Leu)*	1.28	(1.05, 1.71)	<0.0001
19	*Acetone (Ace)*	1.28	(0.85, 1.99)	<0.0001
20	*Creatine (Cre)*	1.26	(0.85, 1.91)	<0.0001
21	*Citrate (Cit)*	1.24	(1.05, 1.48)	<0.0001
22	*2-Hydroxyisobutyrate (2-OH-Isob)*	1.24	(1.07, 1.43)	<0.0001
23	*Isoleucine (Ile)*	1.21	(0.94, 1.49)	<0.0001
24	*4-Hydroxyphenylacetate (4-OH-Phe)*	1.19	(0.97, 1.69)	<0.0001
25	*Formate (For)*	1.16	(0.96, 1.36)	<0.0001
26	*3-Aminoisobutyrate (BAIBA)*	1.15	(0.94, 1.40)	<0.0001
27	*Valine (Val)*	1.14	(0.97, 1.39)	<0.0001
28	*3-Hydroxyisovalerate (3-OH-Isov)*	1.13	(1.02, 1.28)	<0.0001
29	*Gluconate (Glu)*	1.13	(0.92, 1.44)	<0.0001
30	*Tyrosine (Tyr)*	1.12	(0.97, 1.41)	<0.0001
31	Tartrate (Tar)	1.12	(0.74, 1.51)	0.8643
32	*Methylamine (MA)*	1.10	(0.93, 1.41)	<0.0001
33	Dimethylsulfone (DMS)	1.09	(0.87, 1.43)	0.0013
34	Glycolate (Glyc)	1.07	(0.86, 1.27)	0.0066
35	Methanol (Met)	1.07	(0.71, 1.72)	0.1777
36	Urea (Urea)	1.03	(0.87, 1.20)	0.0715
37	Pseudouridine (Pse)	1.03	(0.88, 1.20)	0.1605
38	Dimethylamine (DMA)	1.02	(0.86, 1.21)	0.2783
39	Hypoxanthine (Hyp)	1.00	(0.67, 1.65)	0.4068
40	Uracil (Ura)	0.98	(0.79, 1.22)	0.2208
41	Creatinine (Crea)	0.98	(0.84, 1.18)	0.7036
42	1-Methylnicotinamide (MNA)	0.97	(0.78, 1.24)	0.3221
43	Trimethylamine N-oxide (TMAO)	0.97	(0.79, 1.19)	0.0778
44	*3-Methylxanthine (3-MXan)*	0.90	(0.70, 1.16)	<0.0001
45	*3-Indoxylsulfate (3-Ind)*	0.87	(0.68, 1.04)	<0.0001
46	*Trigonelline (Tri)*	0.73	(0.63, 0.89)	<0.0001
47	*Hippurate (Hip)*	0.70	(0.54, 0.91)	<0.0001

For each metabolite, fold changes (FCs) between normalized post- and pre-exercise concentrations were calculated per participant and presented as median, 25th and 75th percentiles. Significant pre-to-post-exercise differences are shown by false discovery rate (FDR)-corrected *p*-values obtained by Wilcoxon’s signed-rank test. Metabolites are sorted from high to low median FCs. Exercise-responsive metabolites (i.e., metabolites with FDR-adjusted *p*-values < 0.05 and median FCs ≤ or ≥ 1.1, respectively) are indicated in italics.

**Table 3:** Final multiple linear models for the adjusted VO_2peak._

Model	*R* ^2^	*R*^2^ (Adjusted)	BIC	Std.-Beta	95% CI (Lower)	95% CI (Upper)
**Pre**	**0.176**	**0.153**	**300.9**			
*cis*-Aconitate				−0.441	−0.291	−0.106
3-Aminoisobutyrate				0.232	0.047	0.163
*trans*-Aconitate				0.295	0.054	0.212
Tyrosine				−0.213	−0.157	−0.038
Guanidoacetate				−0.208	−0.165	−0.038
Uracil				0.206	0.033	0.156
Lactate				0.230	0.041	0.218
**Post**	**0.081**	**0.070**	**306.6**			
Tyrosine				−0.197	−0.145	−0.034
3-Aminoisobutyrate				0.182	0.028	0.141
1-Methylnicotinamide				−0.149	−0.126	−0.013
**Fold changes**	**0.000**	**0.000**	**311.4**			
(intercept-only model)						

Metabolite variables were selected based on the results of the stepwise regression analyses. All variables were Van der Waerden-transformed prior to analysis and adjusted for age, sex, menopausal status, and LBM. BIC: Bayesian information criterion, CI: confidence interval (of the corresponding regression coefficient in the considered linear model), *R*^2^: coefficient of determination, *R*^2^ (Adjusted): adjusted coefficient of determination, Std.-Beta: Standard-Beta (corresponding regression coefficient using only standardized variables).

## Supplementary Materials

The following are available online at https://www.mdpi.com/2218-1989/10/5/212/s1, Figure S1: Boxplots of urinary metabolite concentrations pre- vs. post-exercise, Figure S2: Boxplots of urinary metabolite fold changes, Figure S3: Score- and loading plots of principal component analyses, File S1: R-script for figure generation, Table S1: Study population and urinary metabolite data, Table S2: Fold changes of urinary metabolites, stratified by sex, Table S3: Correlations between urinary metabolite fold changes, Table S4: Correlations between VO_2peak_ and urinary metabolites, Table S5: Results of the stepwise regression analyses.

## Abbreviations

ATP	adenosine triphosphate
BIC	Bayesian information criterion
CI	confidence interval
CRF	cardiorespiratory fitness
DXA	dual-energy X-ray absorptiometry
FC	fold change
FCs	fold changes
FDR	false discovery rat
HR_max_	maximum heart rate
KarMeN	Karlsruhe Metabolomics and Nutrition
LBM	lean body mass
NMR	nuclear magnetic resonance
NOESY	nuclear overhauser enhancement spectroscopy
*p*	*p*-value of context-dependent test
PC	principal component
PCA	principal component analysis
PCs	principal components
P_IAT_	power at individual anaerobic threshold
P_max_	maximal power
QC	quality control
*r*	Pearson correlation coefficient
*R* ^2^	coefficient of determination
*R*^2^ (adjusted)	adjusted coefficient of determination
SD	standard deviation
TCA	tricarboxylic acid
TSP	trimethylsilylpropanoic acid
VO_2_	oxygen uptake
VO_2max_	maximal oxygen uptake
VO_2peak_	peak oxygen uptake
