# Supplementary material for: An NMR-Based Approach to Identify Urinary Metabolites Associated with Acute Physical Exercise and Cardiorespiratory Fitness in Healthy Humans—Results of the KarMeN Study
Source: Metabolites. 2020 May 21;10(5):212. doi: 10.3390/metabo10050212 (PMC7281079; doi:10.3390/metabo10050212)
Supplement: Supplementary file 1 [file metabolites-10-00212-s001.zip › Supplement-790661_revised/Figure S1_Boxplots of Urinary Metabolite Concentrations Pre- vs. Post-Exercise.pdf]

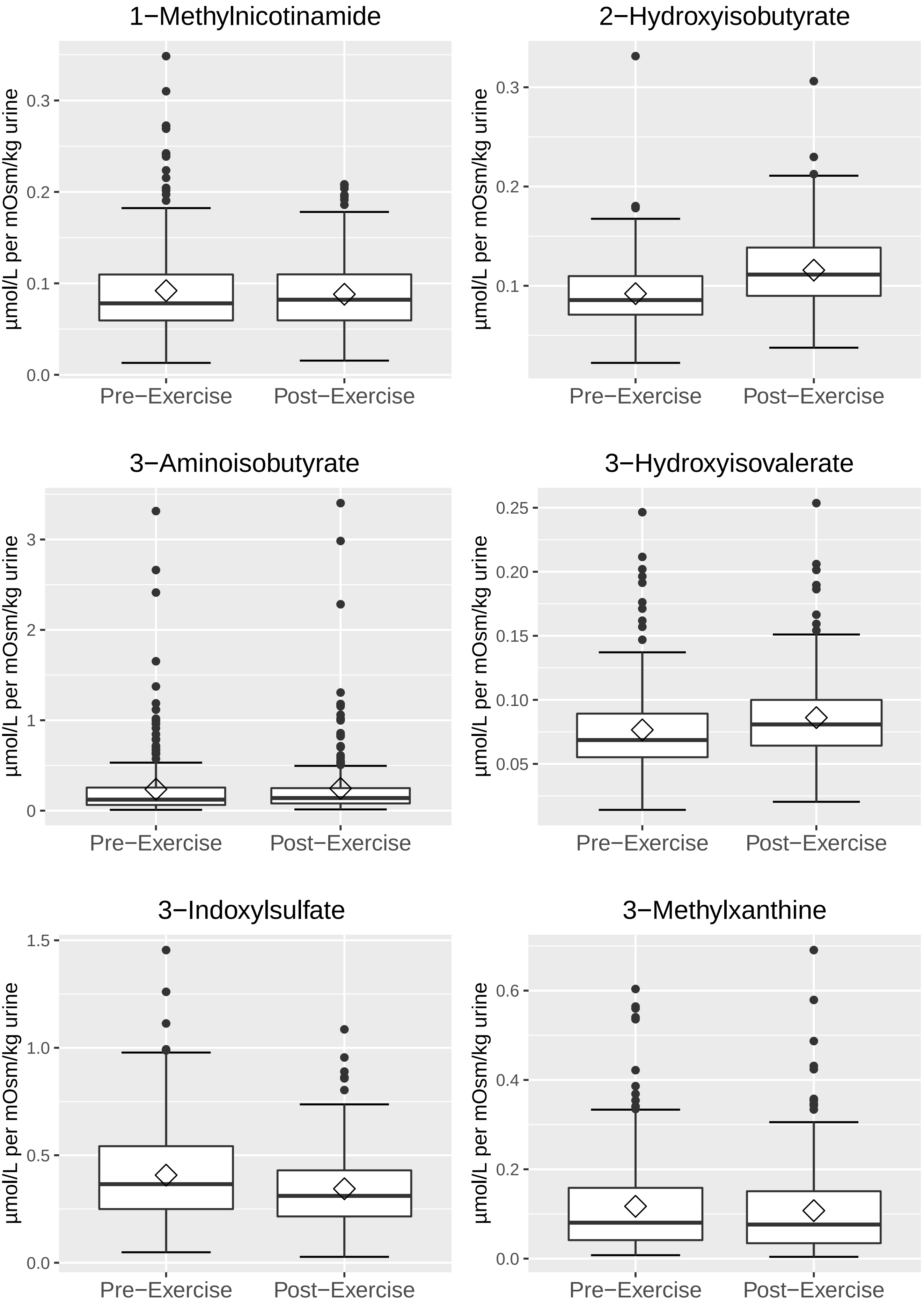
1−Methylnicotinamide
2−Hydroxyisobutyrate
3−Aminoisobutyrate
3−Hydroxyisovalerate
3−Indoxylsulfate
3−Methylxanthine
µmol/L per mOsm/kg urine
0.0
0.1
0.2
0.3
0.05
0.10
0.15
0.20
0.25
0
1
2
3
0.5
1.0
1.5
0.4
0.6
Pre−Exercise
Post−Exercise

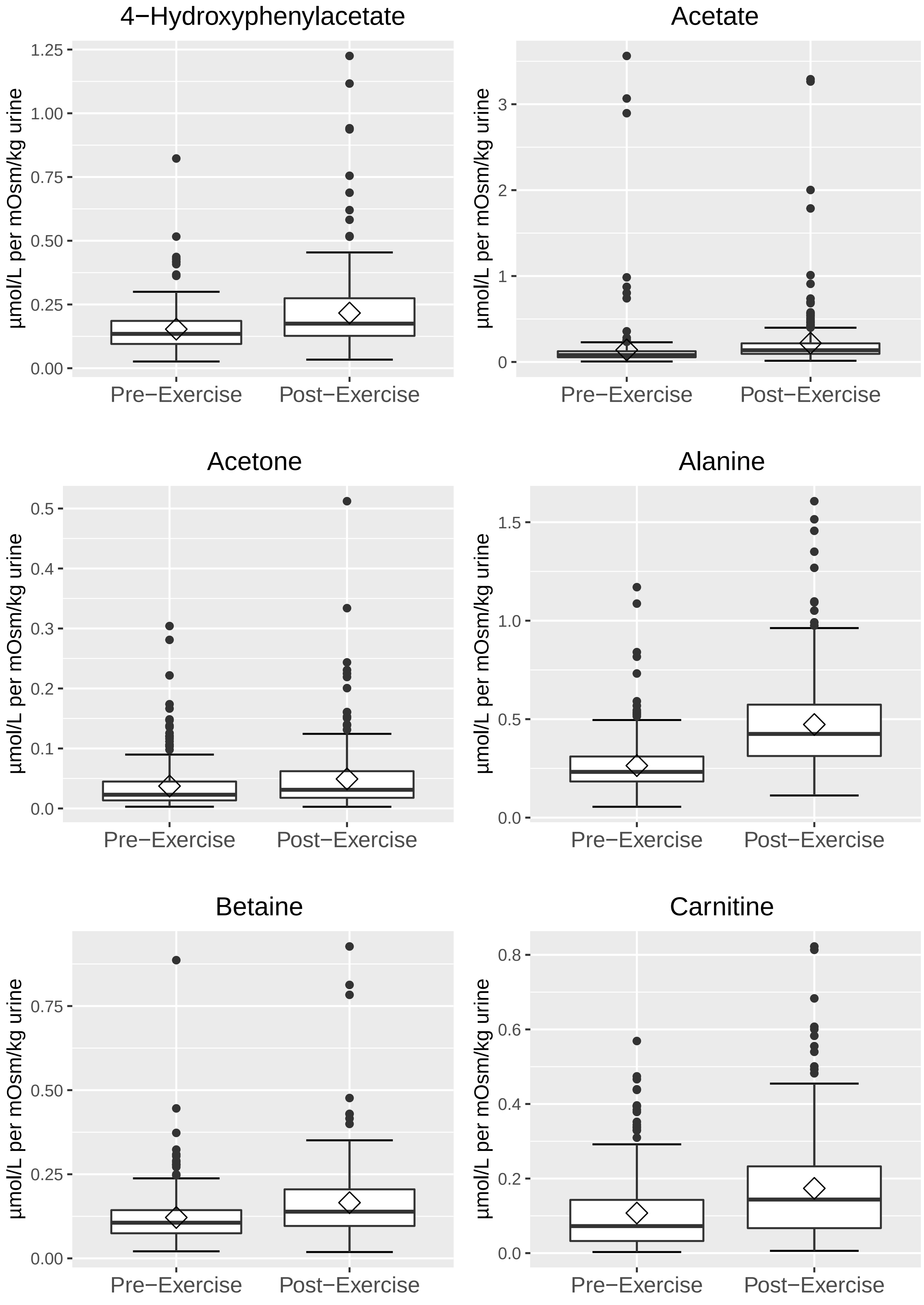
4−Hydroxyphenylacetate
Acetate
Acetone
Alanine
Betaine
Carnitine
µmol/L per mOsm/kg urine
Pre−Exercise
Post−Exercise
0.00
0.25
0.50
0.75
1.00
1.25
0
1
2
3
0.0
0.1
0.2
0.3
0.4
0.5
0.5
1.0
1.5
0.6
0.8

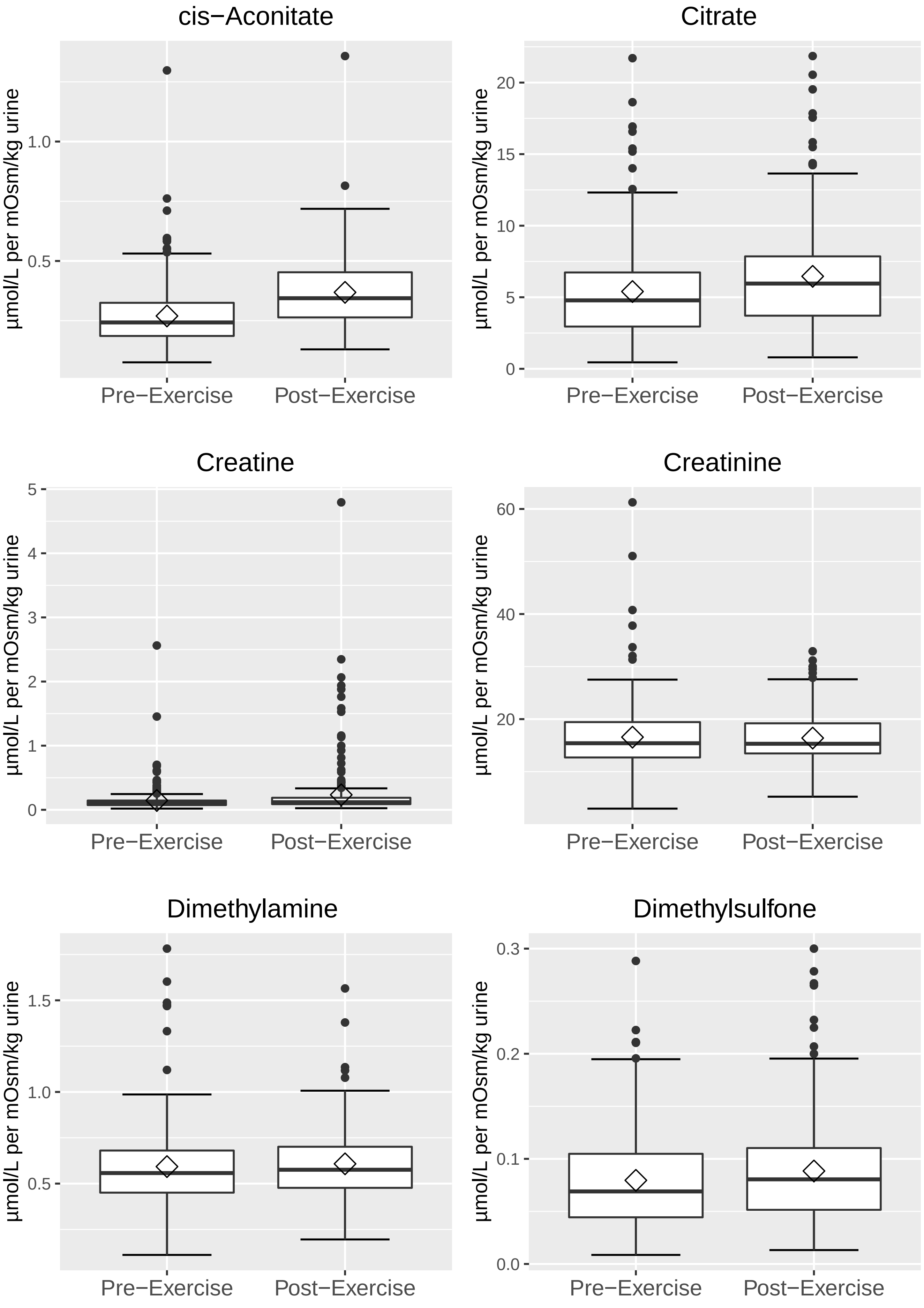
cis−Aconitate
Citrate
Creatine
Creatinine
Dimethylamine
Dimethylsulfone
µmol/L per mOsm/kg urine
Pre−Exercise
Post−Exercise
0.5
1.0
0
5
10
15
20
1
2
3
4
40
60
0.0
0.1
0.2
0.3
1.5

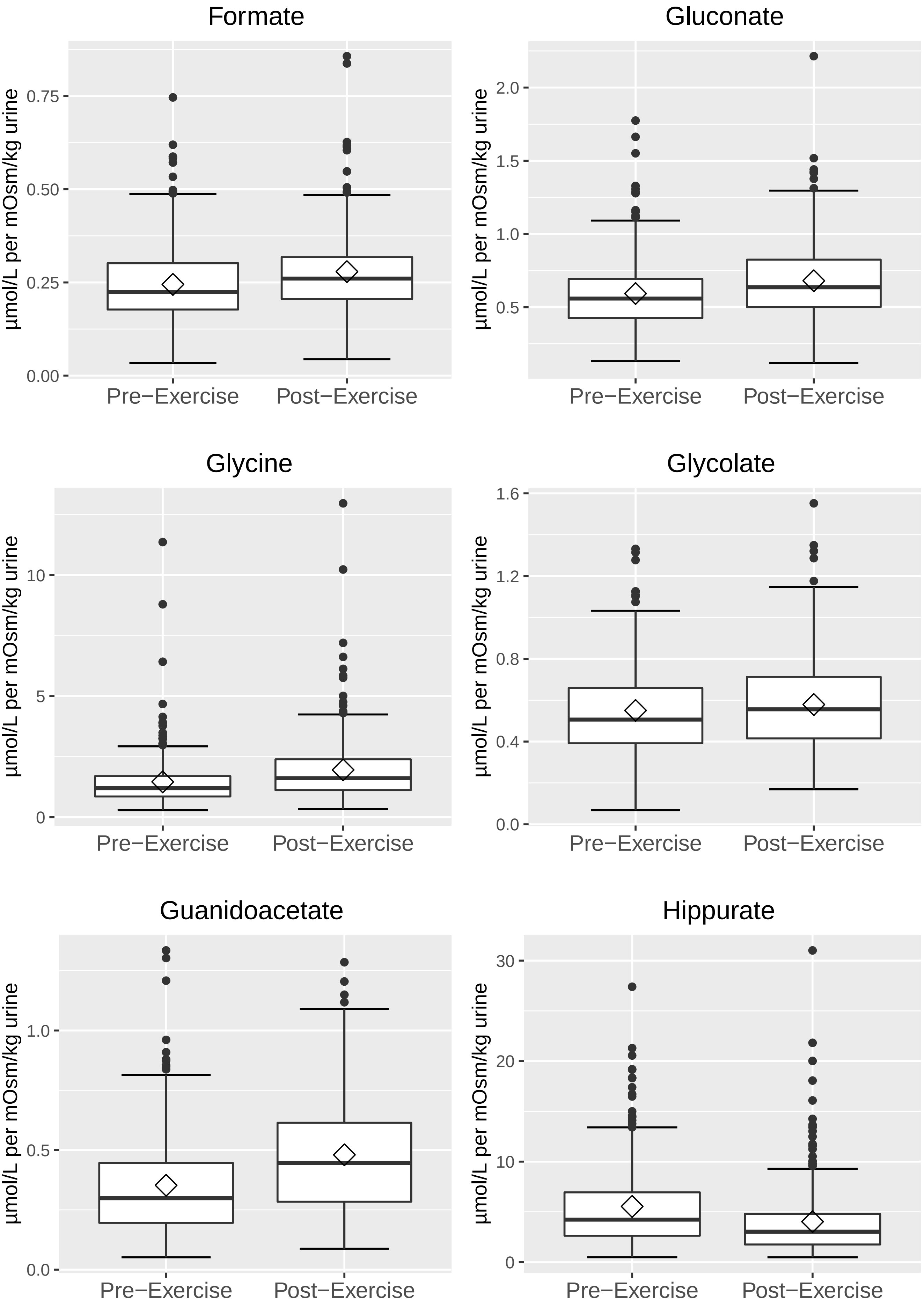
Formate
Gluconate
Glycine
Glycolate
Guanidoacetate
Hippurate
µmol/L per mOsm/kg urine
Pre−Exercise
Post−Exercise
0.00
0.25
0.50
0.75
0.5
1.0
1.5
2.0
0
5
10
0.0
0.4
0.8
1.2
1.6
0.0
0.5
1.0
0
10
20
30

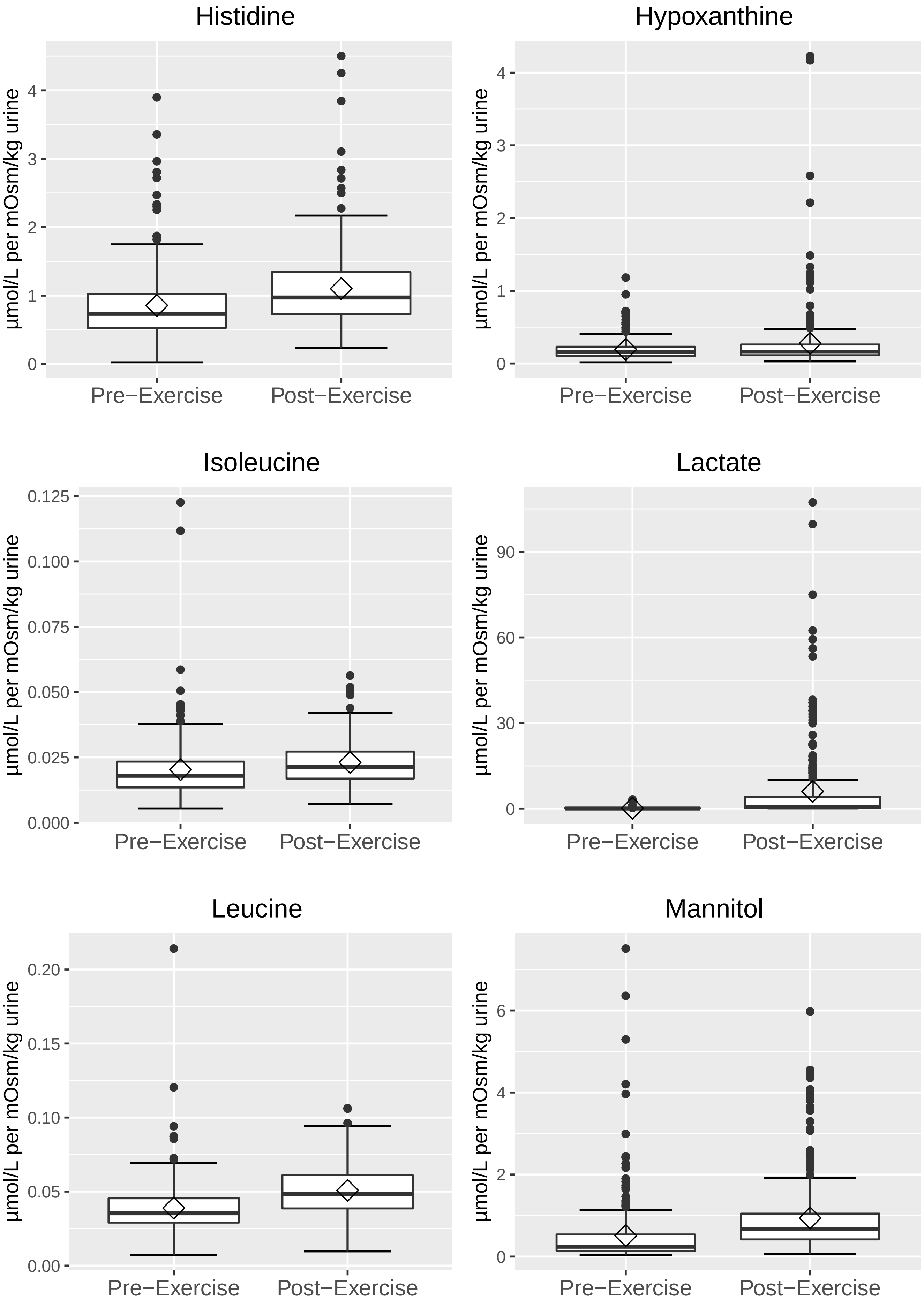
Histidine
μmol/L per mOsm/kg urine
0
1
2
3
4
Pre−Exercise
Post−Exercise
Hypoxanthine
μmol/L per mOsm/kg urine
0
1
2
3
4
Pre−Exercise
Post−Exercise
Isoleucine
μmol/L per mOsm/kg urine
0.000
0.025
0.050
0.075
0.100
0.125
Pre−Exercise
Post−Exercise
Lactate
μmol/L per mOsm/kg urine
0
30
60
90
Pre−Exercise
Post−Exercise
Leucine
μmol/L per mOsm/kg urine
0.00
0.05
0.10
0.15
0.20
Pre−Exercise
Post−Exercise
Mannitol
μmol/L per mOsm/kg urine
0
2
4
6
Pre−Exercise
Post−Exercise

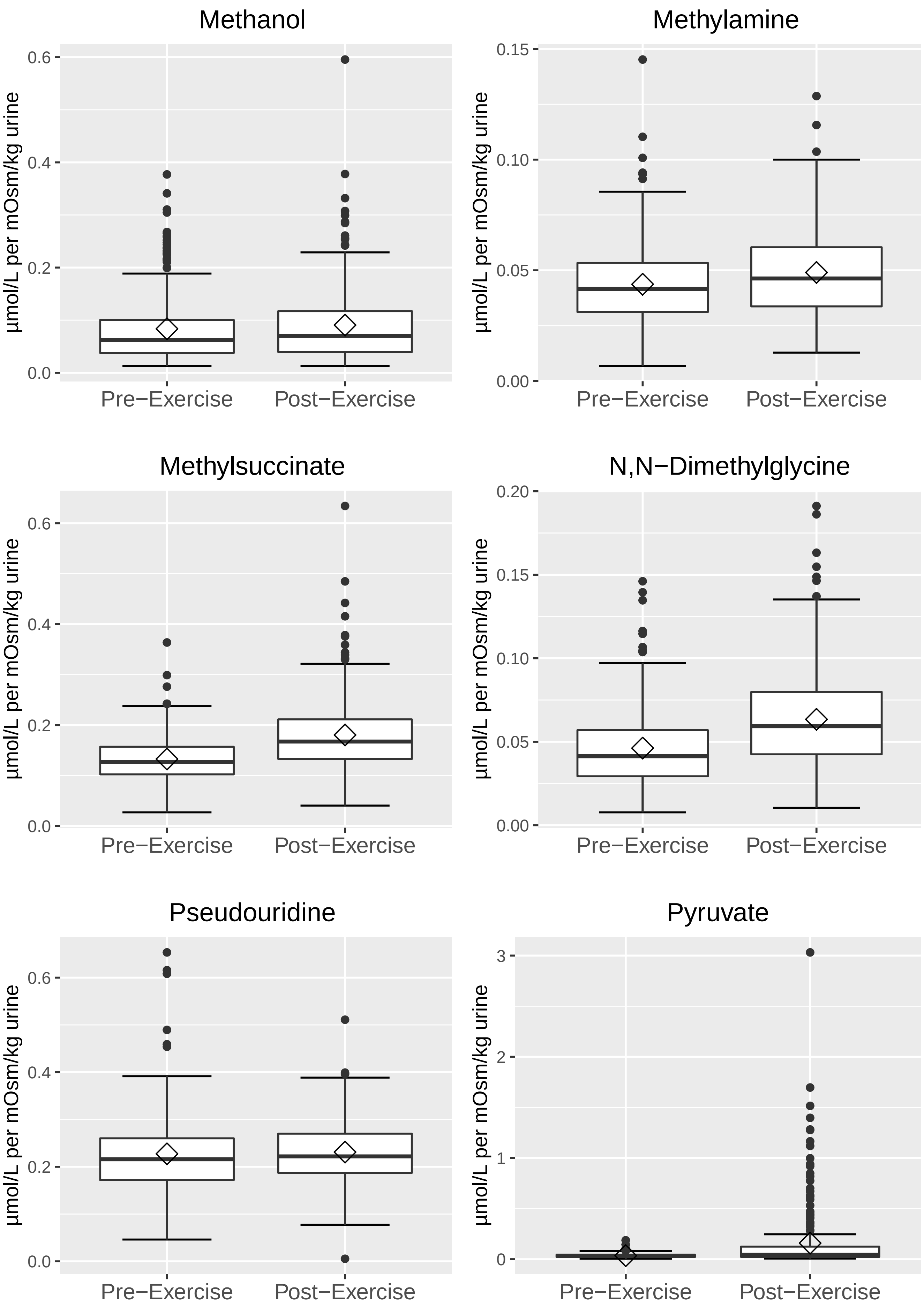
Methanol
Methylamine
Methylsuccinate
N,N−Dimethylglycine
Pseudouridine
Pyruvate
µmol/L per mOsm/kg urine
Pre−Exercise
Post−Exercise
0.0
0.2
0.4
0.6
0.00
0.05
0.10
0.15
0.20
0
1
2
3

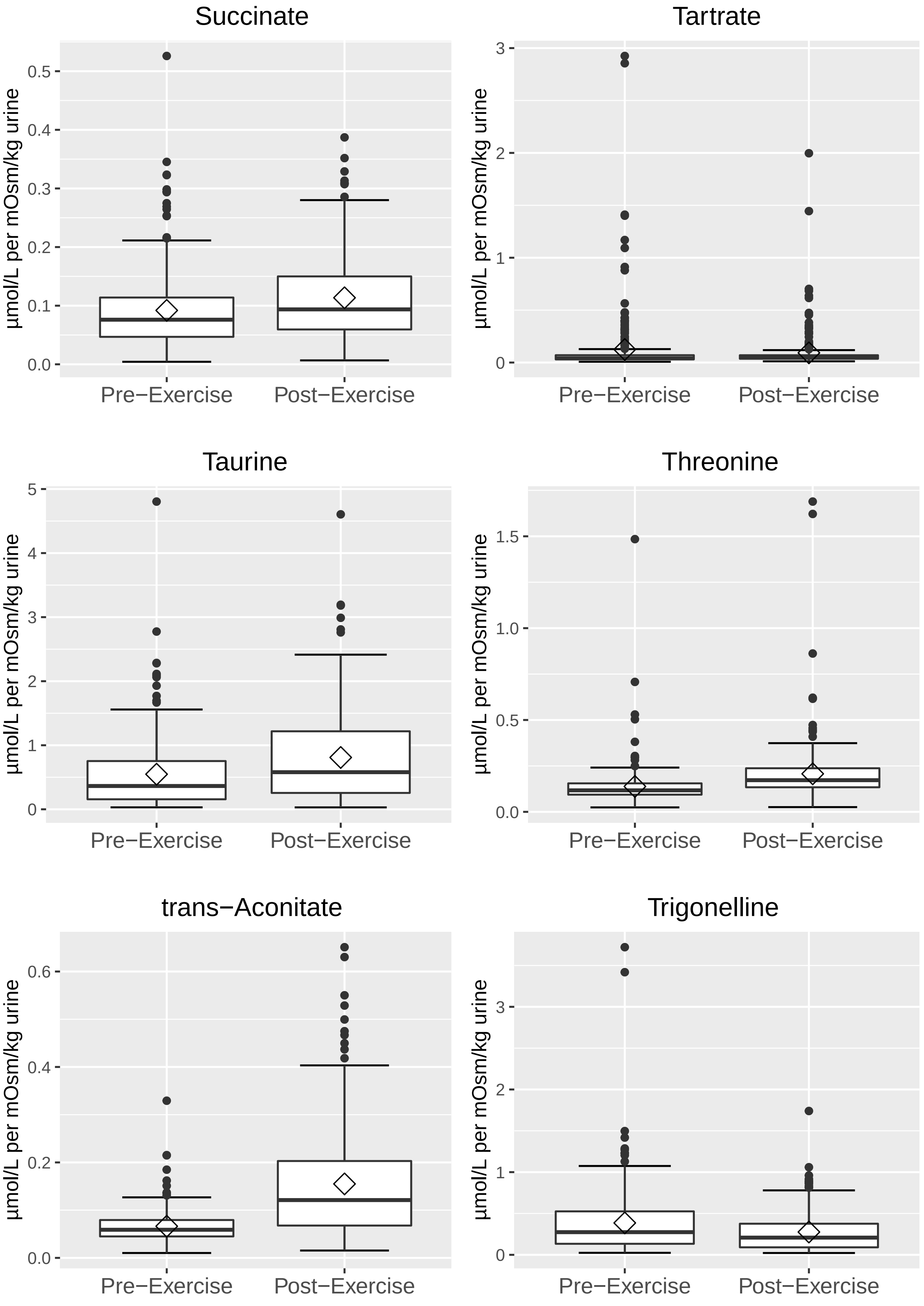

Succinate
Tartrate
Taurine
Threonine
trans−Aconitate
Trigonelline
µmol/L per mOsm/kg urine
Pre−Exercise
Post−Exercise
0.0
0.1
0.2
0.3
0.4
0.5
0
1
2
3
4
5
0.5
1.0
1.5
0.6

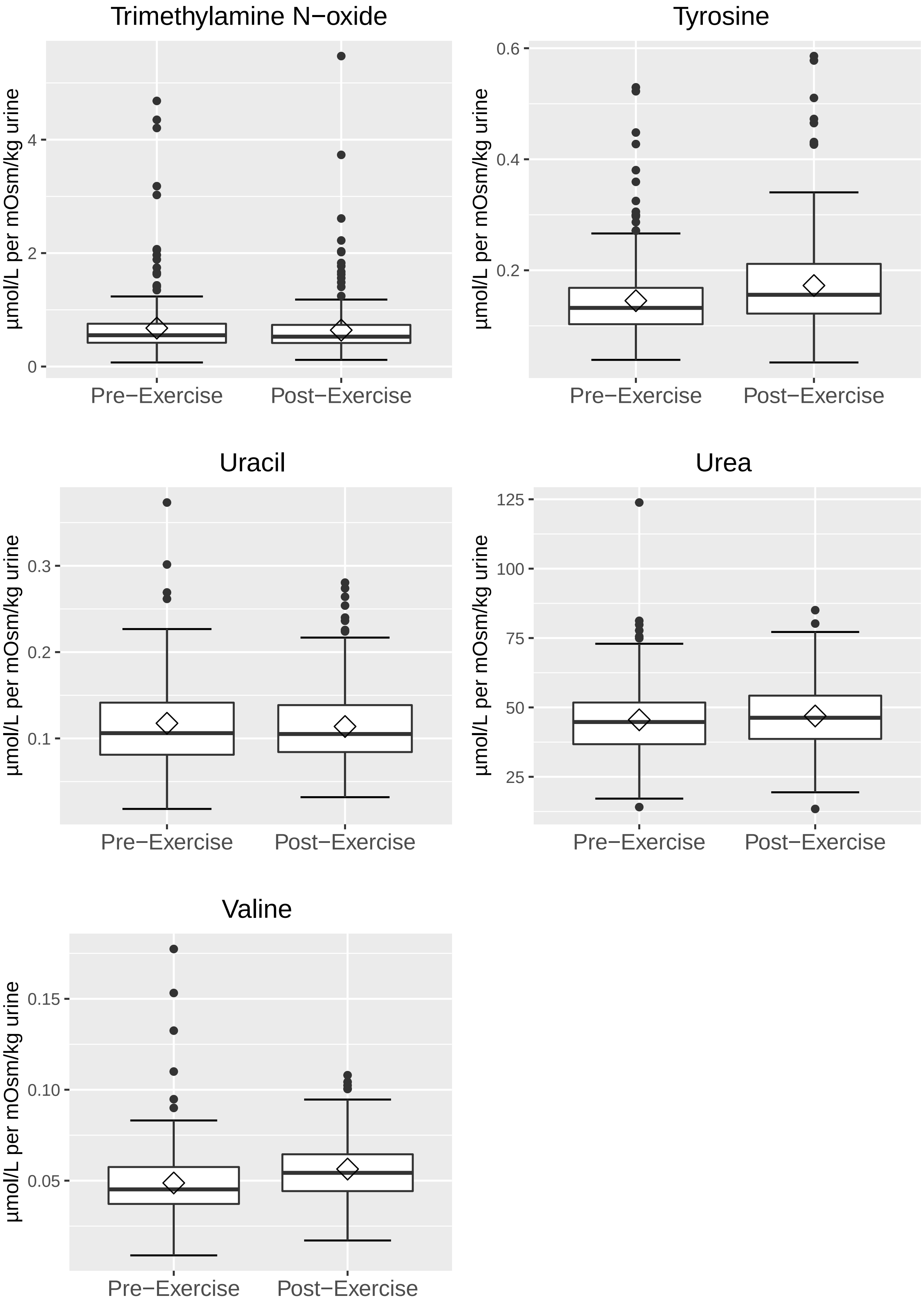

Trimethylamine N−oxide
μmol/L per mOsm/kg urine
0
2
4
Pre−Exercise
Post−Exercise
Tyrosine
μmol/L per mOsm/kg urine
0.2
0.4
0.6
Pre−Exercise
Post−Exercise
Uracil
μmol/L per mOsm/kg urine
0.1
0.2
0.3
Pre−Exercise
Post−Exercise
Urea
μmol/L per mOsm/kg urine
25
50
75
100
125
Pre−Exercise
Post−Exercise
Valine
μmol/L per mOsm/kg urine
0.05
0.10
0.15
Pre−Exercise
Post−Exercise
