# Supplementary material for: An NMR-Based Approach to Identify Urinary Metabolites Associated with Acute Physical Exercise and Cardiorespiratory Fitness in Healthy Humans—Results of the KarMeN Study
Source: Metabolites. 2020 May 21;10(5):212. doi: 10.3390/metabo10050212 (PMC7281079; doi:10.3390/metabo10050212)
Supplement: Supplementary file 1 [file metabolites-10-00212-s001.zip › Supplement-790661_revised/Figure S2_Boxplots of Urinary Metabolite Fold Changes.pdf]

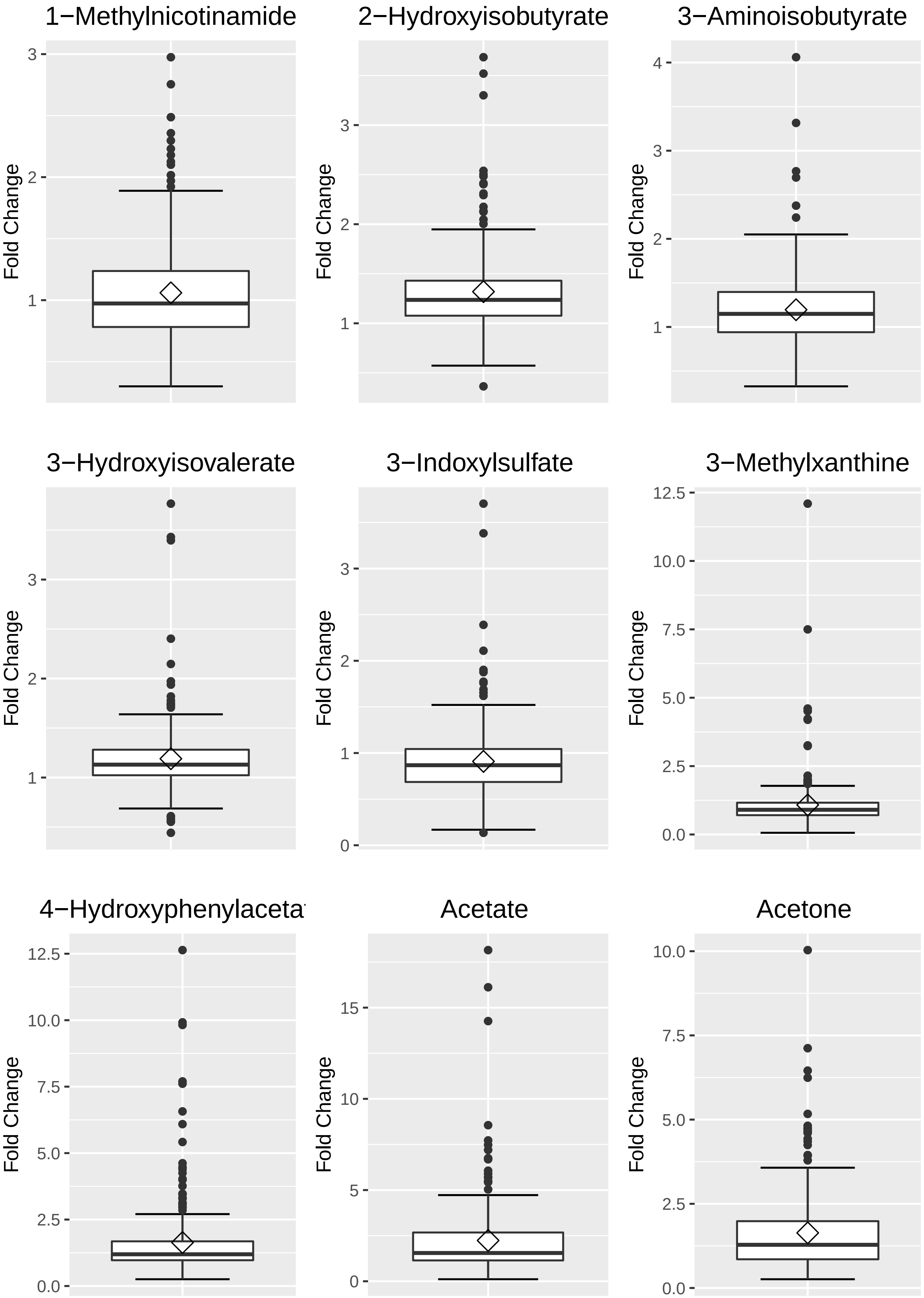
1−Methylnicotinamide
Fold Change
3
2
1
2−Hydroxyisobutyrate
Fold Change
3
2
1
3−Aminoisobutyrate
Fold Change
4
3
2
1
3−Hydroxyisovalerate
Fold Change
3
2
1
3−Indoxylsulfate
Fold Change
3
2
1
0
3−Methylxanthine
Fold Change
12.5
10.0
7.5
5.0
2.5
0.0
4−Hydroxyphenylacetat
Fold Change
12.5
10.0
7.5
5.0
2.5
0.0
Acetate
Fold Change
15
10
5
0
Acetone
Fold Change
10.0
7.5
5.0
2.5
0.0

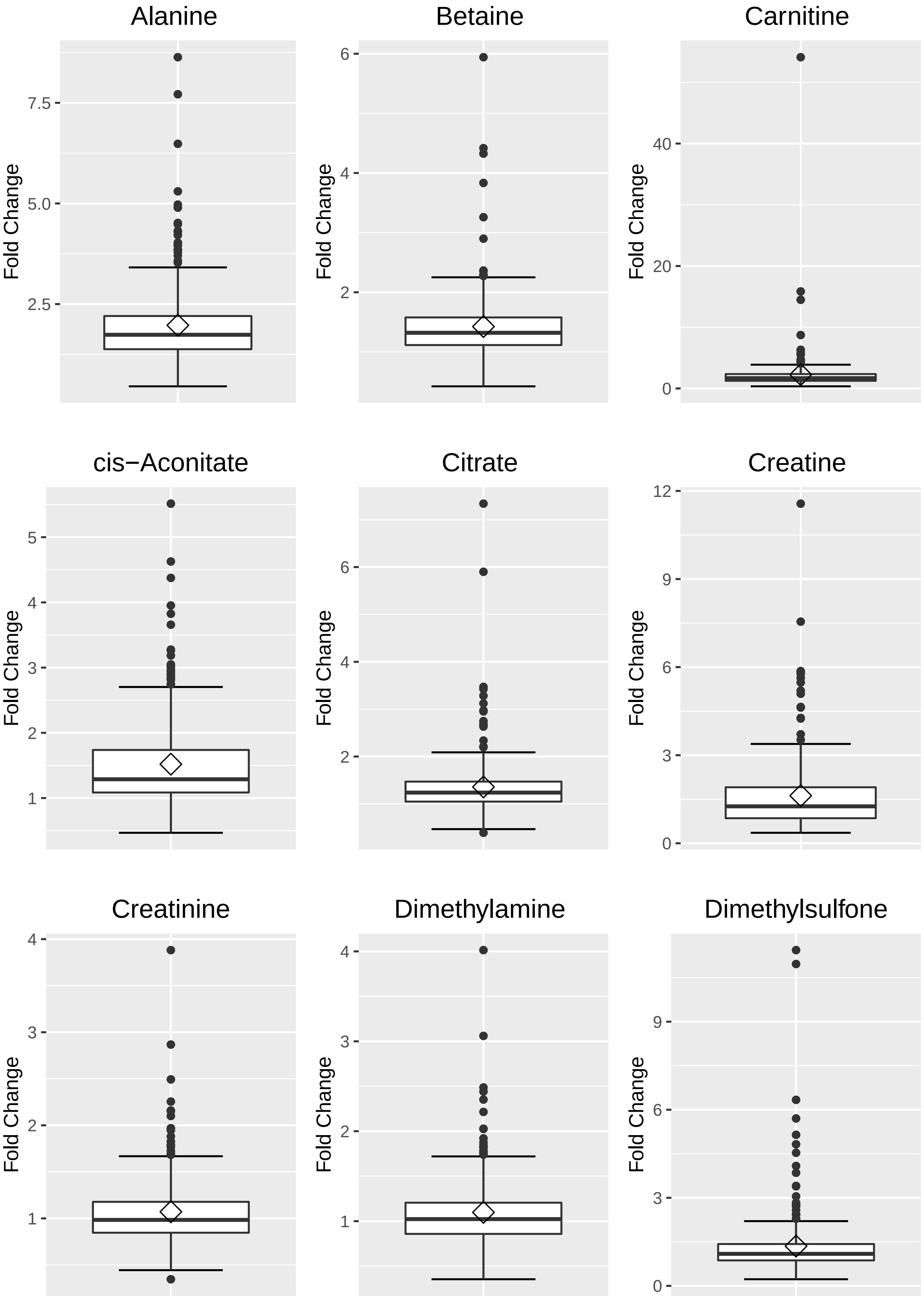

Alanine
Betaine
Carnitine
cis−Aconitate
Citrate
Creatine
Creatinine
Dimethylamine
Dimethylsulfone
Fold Change
2.5
5.0
7.5
2
4
6
0
20
40
1
2
3
4
5
2
4
6
0
3
6
9
12
1
2
3
4
1
2
3
4
0
3
6
9

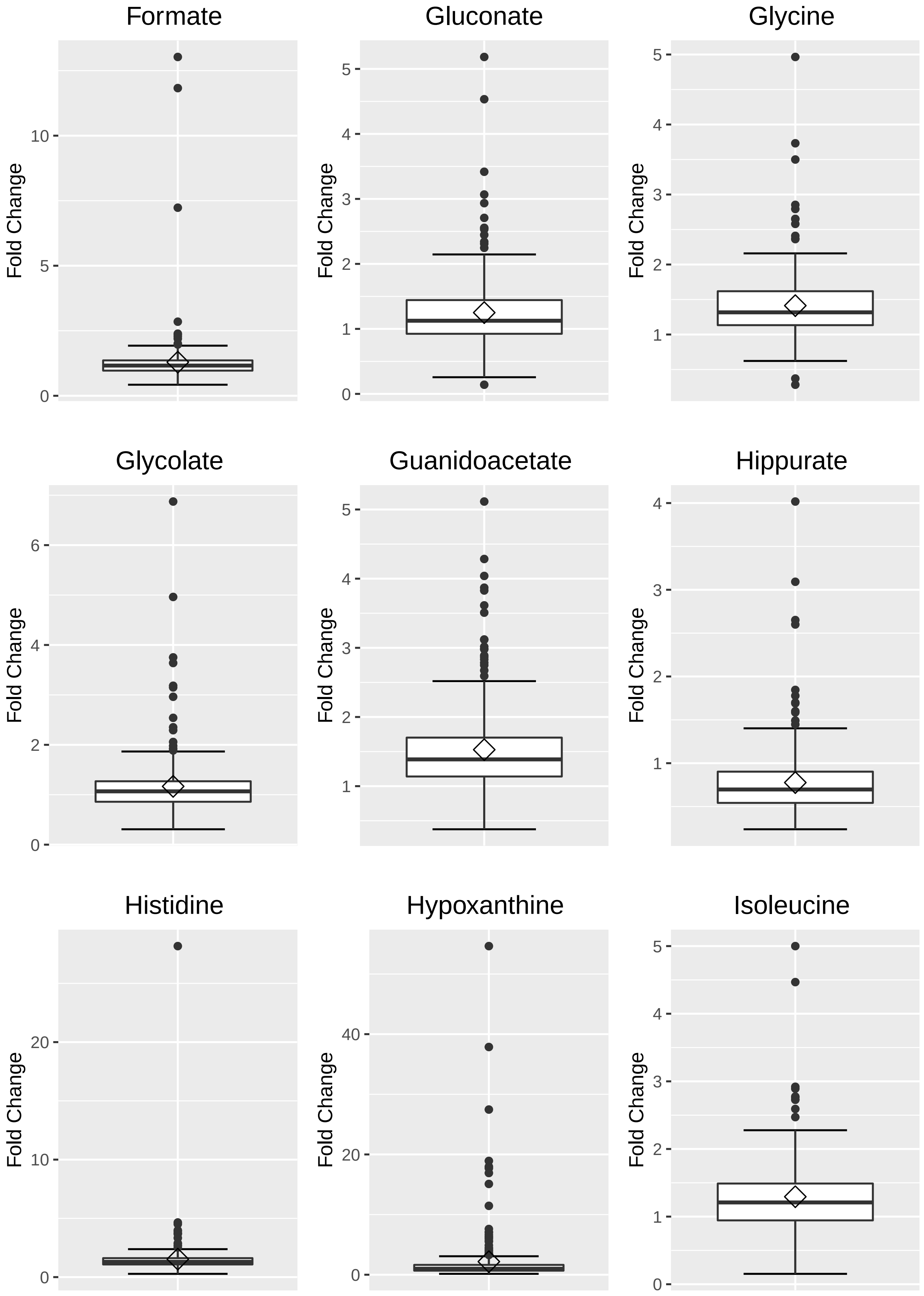
Formate
Fold Change
0
5
10
Gluconate
Fold Change
0
1
2
3
4
5
Glycine
Fold Change
1
2
3
4
5
Glycolate
Fold Change
0
2
4
6
Guanidoacetate
Fold Change
1
2
3
4
5
Hippurate
Fold Change
1
2
3
4
Histidine
Fold Change
0
10
20
Hypoxanthine
Fold Change
0
20
40
Isoleucine
Fold Change
0
1
2
3
4
5

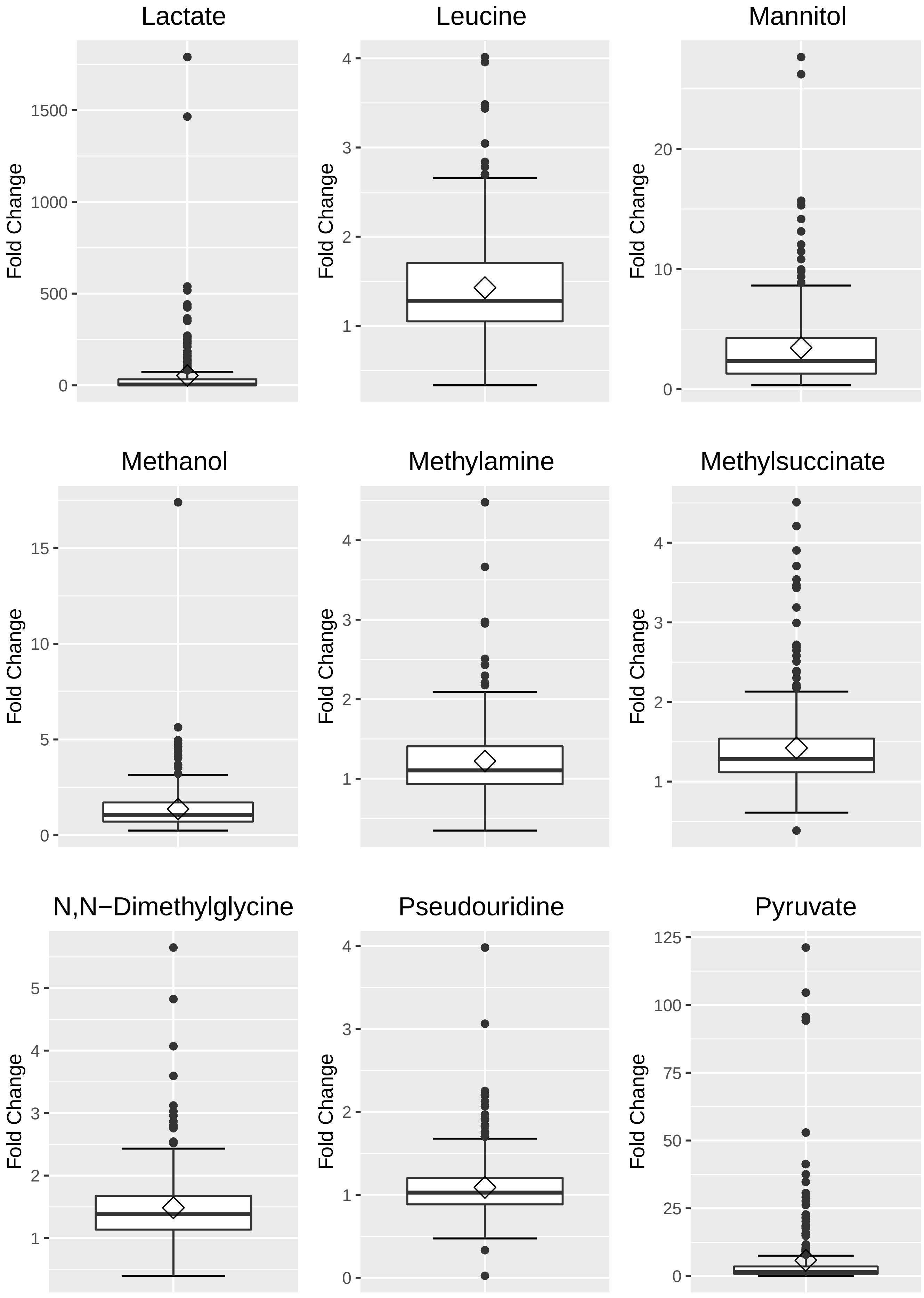
Lactate
Leucine
Mannitol
Methanol
Methylamine
Methylsuccinate
N,N−Dimethylglycine
Pseudouridine
Pyruvate
Fold Change
0
500
1000
1500
1
2
3
4
10
20
5
15
25
50
75
100
125

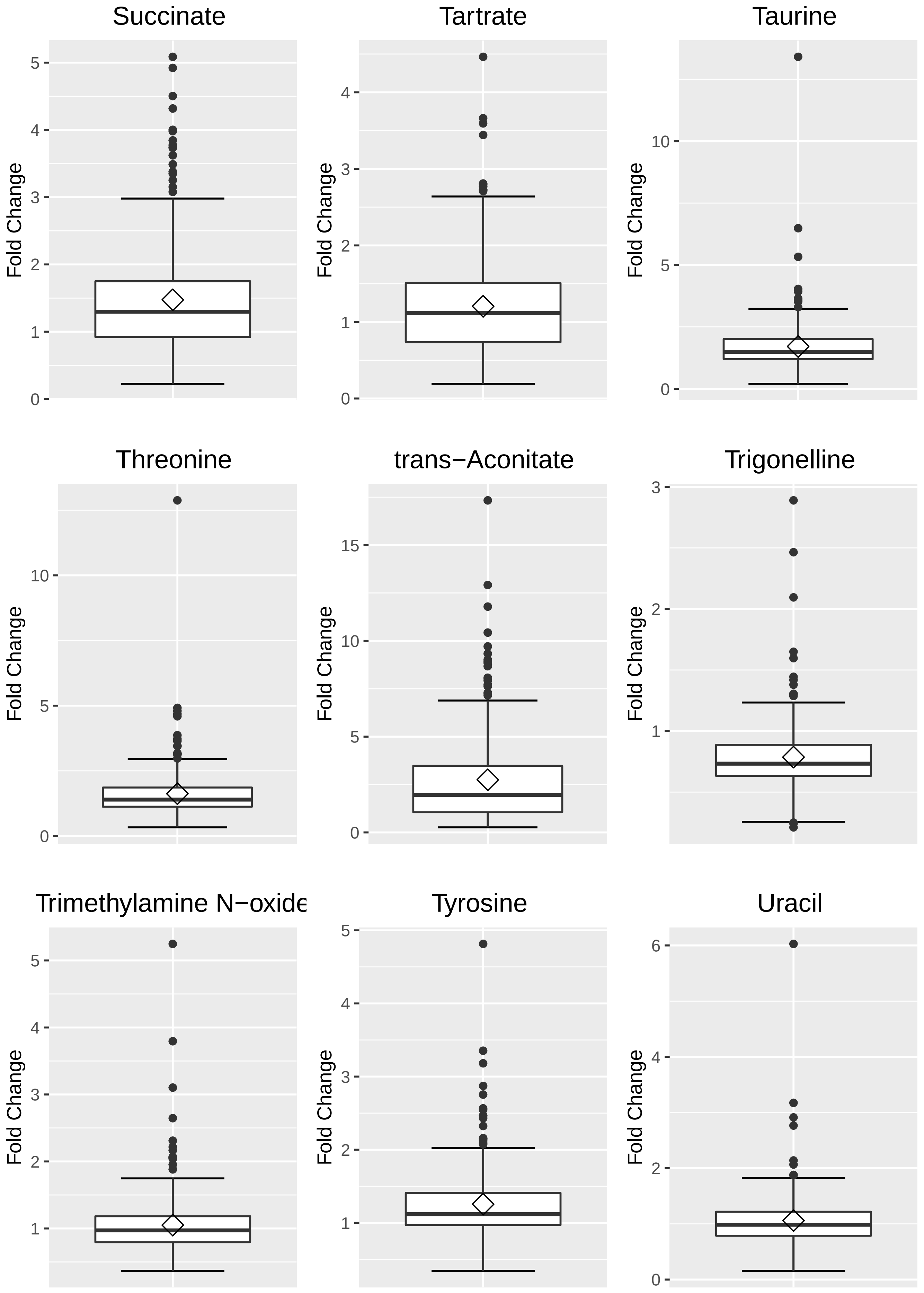
Succinate
Fold Change
0
1
2
3
4
5
Tartrate
Fold Change
0
1
2
3
4
Taurine
Fold Change
0
5
10
Threonine
Fold Change
0
5
10
trans−Aconitate
Fold Change
0
5
10
15
Trigonelline
Fold Change
1
2
3
Trimethylamine N−oxide
Fold Change
1
2
3
4
5
Tyrosine
Fold Change
1
2
3
4
5
Uracil
Fold Change
0
2
4
6

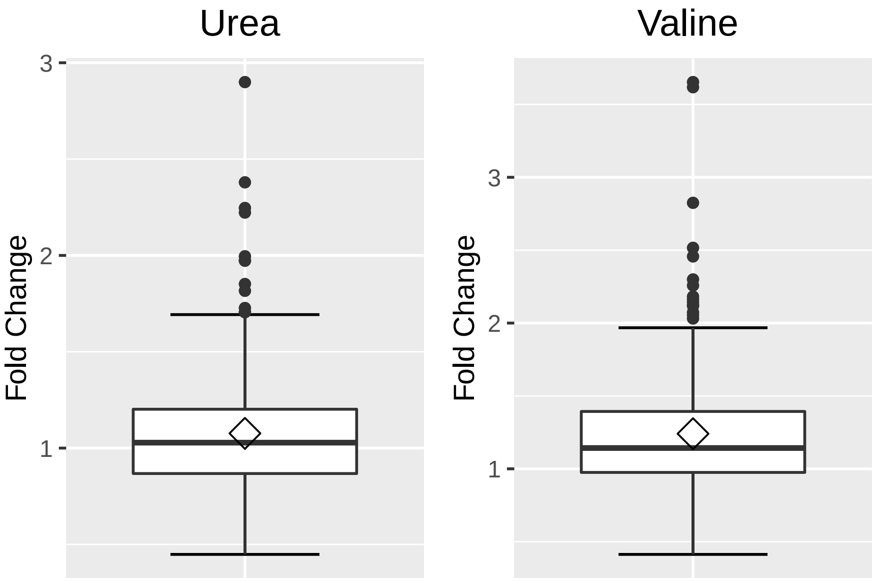

Urea
Valine
Fold Change
Fold Change
3
2
1
3
2
1
