# Supplementary material for: An NMR-Based Approach to Identify Urinary Metabolites Associated with Acute Physical Exercise and Cardiorespiratory Fitness in Healthy Humans—Results of the KarMeN Study
Source: Metabolites. 2020 May 21;10(5):212. doi: 10.3390/metabo10050212 (PMC7281079; doi:10.3390/metabo10050212)
Supplement: Supplementary file 1 [file metabolites-10-00212-s001.zip › Supplement-790661_revised/Figure S3_Score- and Loading Plots of Principal Component Analyses.pdf]

Figure S3. Score- and Loading Plots of Principal Component Analyses

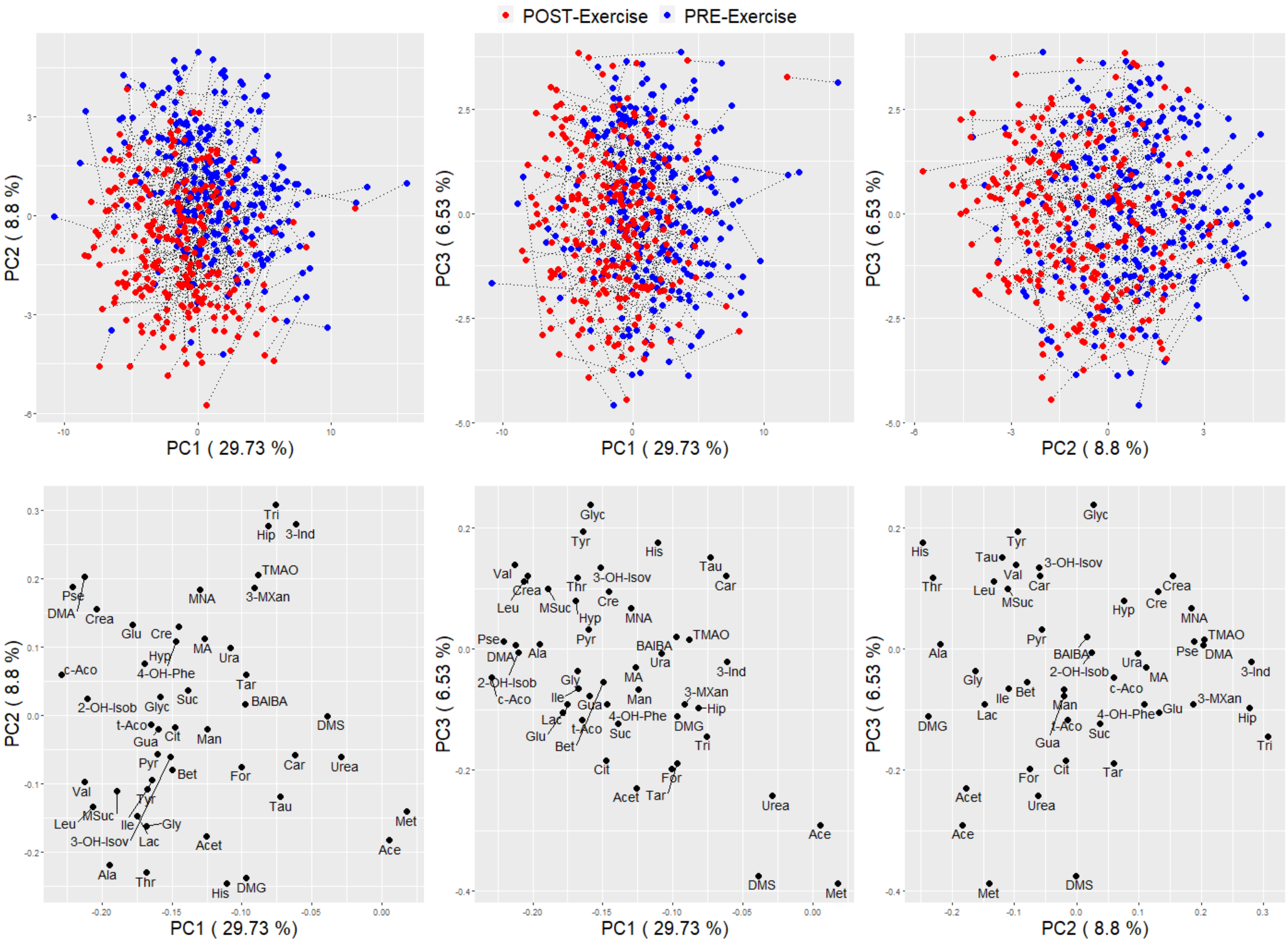

Figure S3a. PCA Score and Loading Plots of a Combined Pre- and Post-Exercise Urinary Metabolite Data Matrix Containing 2x255 Participants and 47 Metabolites. The first three PCs are visualized; Top: Score Plots, data points stand for participants and are color coded according to the pre- or post-exercise state; Bottom: Loading Plots, data points stand for metabolites.

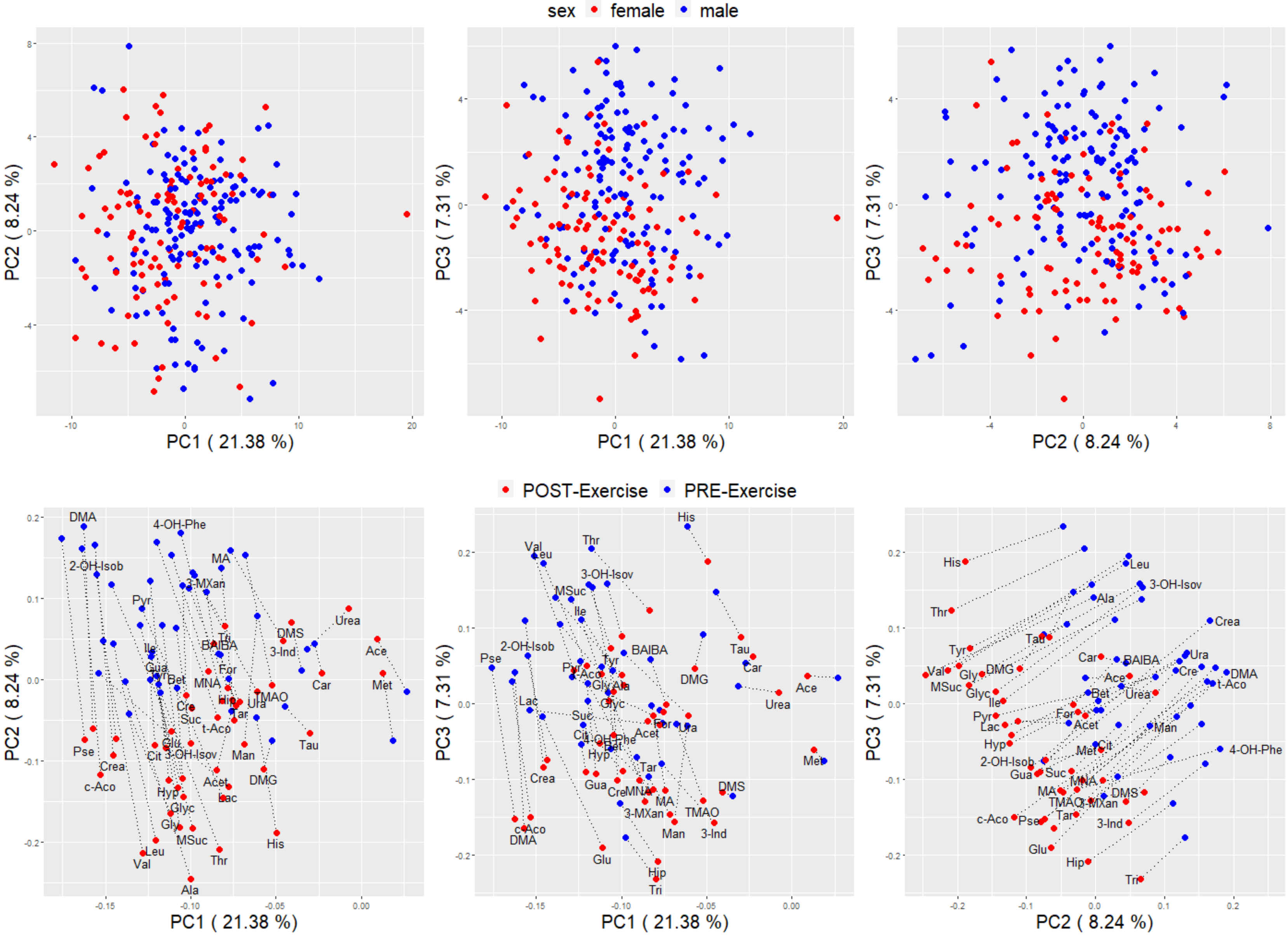

Figure S3b. PCA Score and Loading Plots of a Combined Pre- and Post-Exercise Urinary Metabolite Data Matrix Containing 255 Participants and 2x47 Metabolites. The first three PCs are visualized; Top: Score Plots, data points stand for participants and are color coded according to the sex of the participants; Bottom: Loading Plots, data points stand for metabolites and are color coded according to the pre- or post-exercise state.

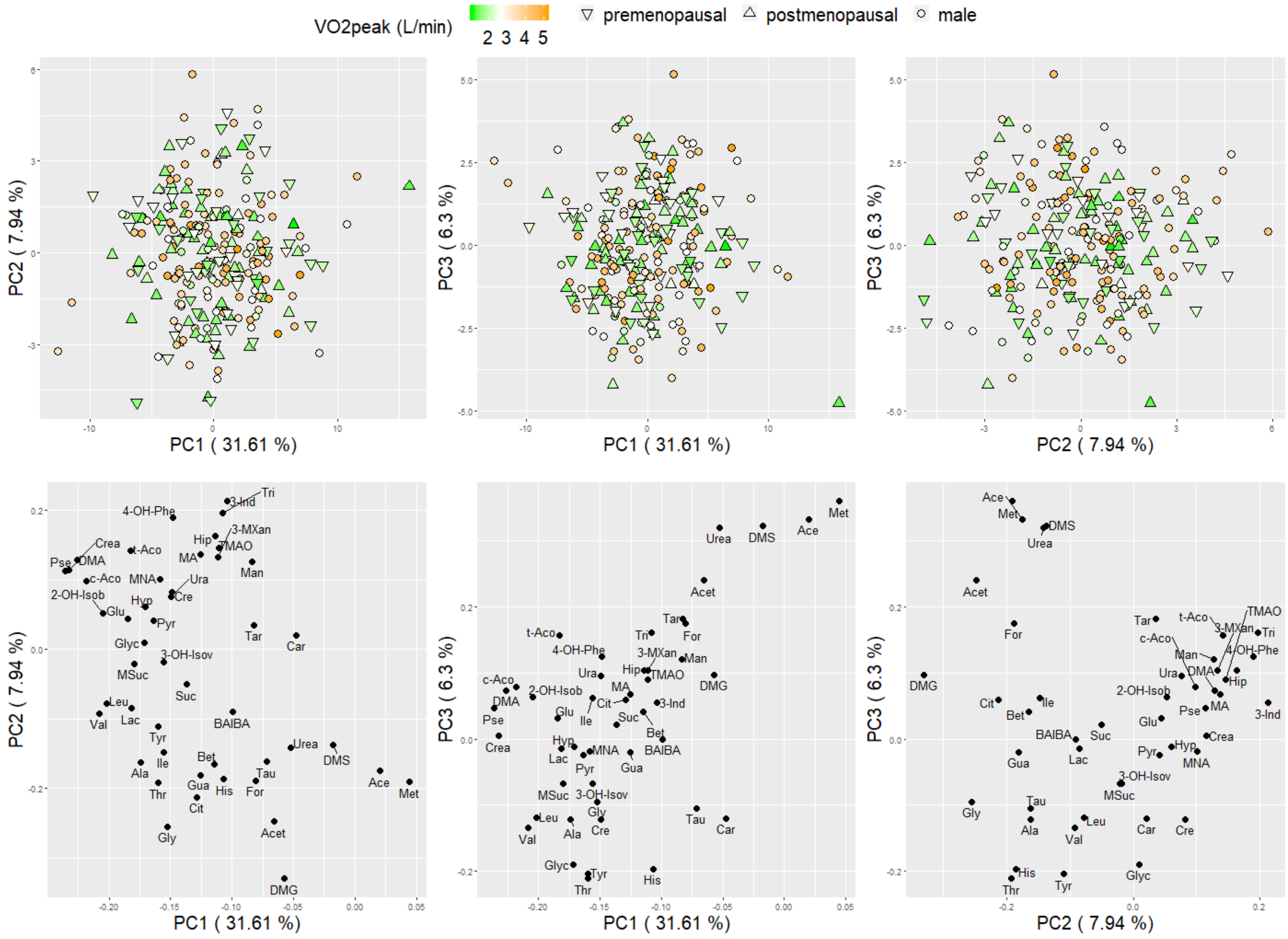

Figure S3c. PCA Score and Loading Plots of the Adjusted Pre-Exercise Urinary Metabolite Data. The first three PCs are visualized; Top: Score Plots, data points stand for participants and are color coded according to the VO_{2peak} of the participants; Bottom: Loading Plots, data points stand for metabolites.

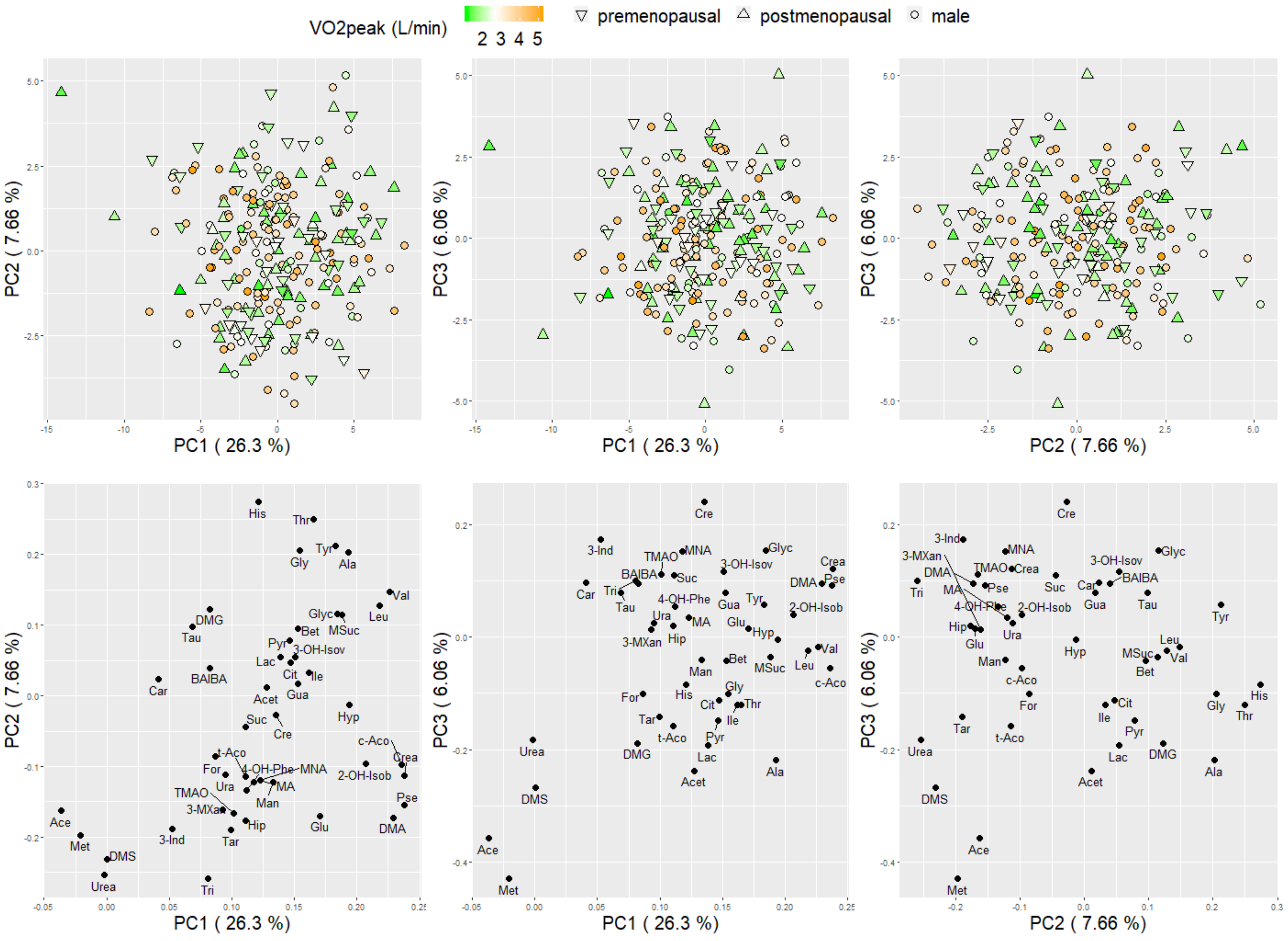

Figure S3d. PCA Score and Loading Plots of the Adjusted Post-Exercise Urinary Metabolite Data. The first three PCs are visualized; Top: Score Plots, data points stand for participants and are color coded according to the VO_{2peak} of the participants; Bottom: Loading Plots, data points stand for metabolites.

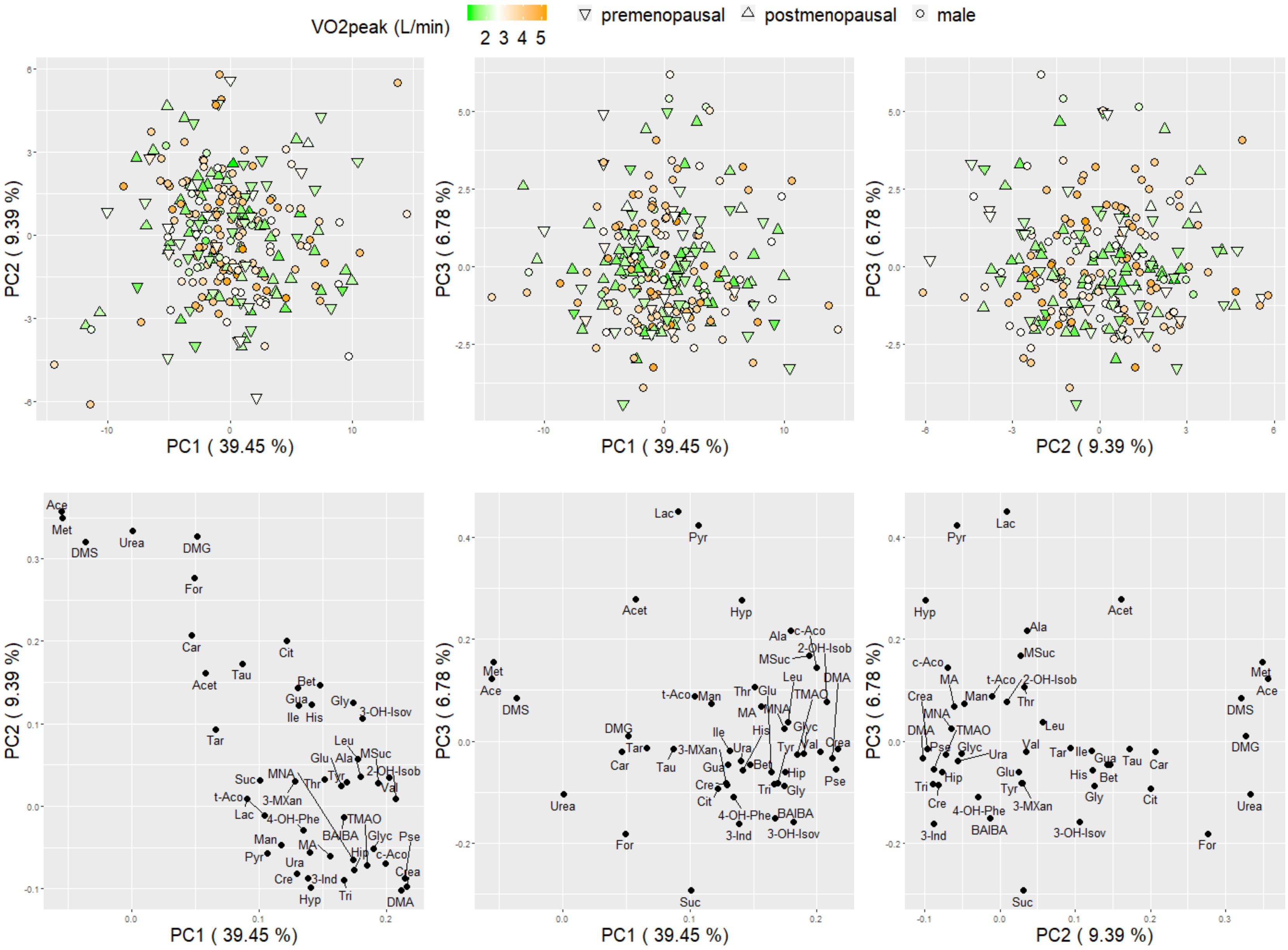

Figure S3e. PCA Score and Loading Plots of the Adjusted Data on Urinary Metabolite Fold Changes. The first three PCs are visualized; Top: Score Plots, data points stand for participants and are color coded according to the VO_{2peak} of the participants; Bottom: Loading Plots, data points stand for metabolites.
