# Supplementary material for: An NMR-Based Approach to Identify Urinary Metabolites Associated with Acute Physical Exercise and Cardiorespiratory Fitness in Healthy Humans—Results of the KarMeN Study
Source: Metabolites. 2020 May 21;10(5):212. doi: 10.3390/metabo10050212 (PMC7281079; doi:10.3390/metabo10050212)
Supplement: Supplementary file 1 [file metabolites-10-00212-s001.zip › Supplement-790661_revised/Table S2_Fold Changes of Urinary Metabolites, Stratified by Sex.pdf]

Table S2. Fold Changes of Urinary Metabolites, Stratified by Sex

Table S2a. Metabolite Fold Changes, stratified by sex

Metabolites	Men<br>Median (25th and 75th percentiles)		Women<br>Median (25th and 75th percentiles)		p-values<br>(FDR-corrected)
1-Methylnicotinamide	0.94	(0.74, 1.20)	1.02	(0.81, 1.30)	0.4065
2-Hydroxyisobutyrate	1.23	(1.07, 1.36)	1.25	(1.10, 1.52)	0.5860
3-Aminoisobutyrate	1.17	(0.92, 1.41)	1.14	(0.94, 1.39)	0.8956
3-Hydroxyisovalerate	1.13	(1.03, 1.32)	1.13	(0.99, 1.26)	0.8272
3-Indoxylsulfate	0.90	(0.68, 1.02)	0.84	(0.68, 1.08)	0.8956
3-Methylxanthine	0.90	(0.70, 1.10)	0.90	(0.70, 1.20)	0.8272
4-Hydroxyphenylacetate	1.18	(0.96, 1.56)	1.24	(0.97, 1.94)	0.8272
Acetate	1.56	(1.16, 2.70)	1.51	(1.11, 2.65)	0.8272
Acetone	1.15	(0.80, 1.80)	1.47	(0.96, 2.38)	0.0504
Alanine	1.73	(1.40, 2.14)	1.76	(1.36, 2.41)	0.8956
Betaine	1.31	(1.10, 1.55)	1.32	(1.12, 1.62)	0.8272
Carnitine	1.67	(1.31, 2.27)	1.69	(1.20, 2.45)	0.9035
cis-Aconitate	1.25	(1.08, 1.58)	1.37	(1.11, 1.92)	0.4065
Citrate	1.29	(1.10, 1.54)	1.16	(0.96, 1.34)	**0.0129**
Creatine	1.16	(0.74, 1.86)	1.42	(1.00, 2.21)	0.1340
Creatinine	0.97	(0.84, 1.14)	0.99	(0.85, 1.23)	0.8272
Dimethylamine	1.02	(0.85, 1.14)	1.04	(0.88, 1.30)	0.4081
Dimethylsulfone	1.05	(0.88, 1.36)	1.10	(0.85, 1.49)	0.8272
Formate	1.19	(1.03, 1.38)	1.08	(0.93, 1.31)	0.2582
Gluconate	1.13	(0.94, 1.38)	1.12	(0.86, 1.55)	0.9035
Glycine	1.31	(1.15, 1.68)	1.32	(1.07, 1.58)	0.4506
Glycolate	1.05	(0.86, 1.26)	1.08	(0.91, 1.30)	0.7141
Guanidoacetate	1.42	(1.13, 1.74)	1.36	(1.16, 1.63)	0.8272
Hippurate	0.69	(0.55, 0.84)	0.71	(0.53, 0.99)	0.8272
Histidine	1.25	(1.07, 1.61)	1.36	(1.11, 1.65)	0.8272
Hypoxanthine	1.03	(0.67, 1.61)	0.96	(0.69, 1.67)	0.8899
Isoleucine	1.21	(0.95, 1.48)	1.23	(0.94, 1.60)	0.8272
Lactate	3.54	(1.47, 53.87)	5.94	(1.93, 20.31)	0.8377
Leucine	1.32	(1.07, 1.67)	1.27	(1.02, 1.77)	0.8272
Mannitol	2.04	(1.21, 3.76)	2.96	(1.57, 5.63)	0.0610
Methanol	1.04	(0.71, 1.54)	1.09	(0.67, 1.84)	0.8272
Methylamine	1.08	(0.89, 1.37)	1.18	(0.97, 1.44)	0.4065
Methylsuccinate	1.26	(1.11, 1.54)	1.30	(1.12, 1.59)	0.8272
N,N-Dimethylglycine	1.36	(1.13, 1.68)	1.40	(1.15, 1.64)	0.8956
Pseudouridine	1.03	(0.88, 1.17)	1.02	(0.90, 1.24)	0.8956
Pyruvate	1.34	(0.85, 3.43)	1.69	(0.92, 5.87)	0.4508
Succinate	1.36	(0.97, 1.78)	1.19	(0.87, 1.74)	0.4065
Tartrate	1.09	(0.74, 1.49)	1.14	(0.72, 1.56)	0.8272
Taurine	1.44	(1.18, 1.93)	1.68	(1.24, 2.15)	0.4065
Threonine	1.42	(1.17, 1.85)	1.36	(1.04, 1.90)	0.5011
trans-Aconitate	1.53	(0.95, 2.82)	2.33	(1.47, 4.44)	**0.0097**
Trigonelline	0.74	(0.65, 0.90)	0.73	(0.60, 0.88)	0.8272
Trimethylamine N-oxide	0.98	(0.77, 1.18)	0.96	(0.80, 1.20)	0.9035
Tyrosine	1.12	(0.98, 1.37)	1.11	(0.96, 1.45)	0.8956
Uracil	0.99	(0.84, 1.26)	0.95	(0.74, 1.16)	0.4065
Urea	1.04	(0.89, 1.19)	1.00	(0.85, 1.23)	0.8272
Valine	1.17	(0.97, 1.38)	1.13	(0.97, 1.47)	0.8956

For each metabolite, fold changes (FCs) between normalized post- and pre-exercise concentrations were calculated per participant and presented as median and 25th and 75th percentiles, stratified by sex. Sex-differences in metabolite FCs were analyzed by Wilcoxon rank-sum test and obtained p-values were FDR-adjusted. Bold p-values indicate significant different metabolite FCs between men and women.

Table S2b. Ranking of Metabolite Fold Changes (sorted from high to low median fold changes), stratified by sex

Men		Women	
**Metabolite**	**Median Fold Change**	**Metabolite**	**Median Fold Change**
Lactate	3.54	Lactate	5.94
Mannitol	2.04	Mannitol	2.96
Alanine	1.73	**trans-Aconitate**	**2.33**
Carnitine	1.67	Alanine	1.76
Acetate	1.56	Carnitine	1.69
**trans-Aconitate**	**1.53**	Pyruvate	1.69
Taurine	1.44	Taurine	1.68
Threonine	1.42	Acetate	1.51
Guanidoacetate	1.42	Acetone	1.47
Succinate	1.36	Creatine	1.42
N,N-Dimethylglycine	1.36	N,N-Dimethylglycine	1.40
Pyruvate	1.34	cis-Aconitate	1.37
Leucine	1.32	Guanidoacetate	1.36
Glycine	1.31	Histidine	1.36
Betaine	1.31	Threonine	1.36
**Citrate**	**1.29**	Glycine	1.32
Methylsuccinate	1.26	Betaine	1.32
cis-Aconitate	1.25	Methylsuccinate	1.30
Histidine	1.25	Leucine	1.27
2-Hydroxyisobutyrate	1.23	2-Hydroxyisobutyrate	1.25
Isoleucine	1.21	4-Hydroxyphenylacetate	1.24
Formate	1.19	Isoleucine	1.23
4-Hydroxyphenylacetate	1.18	Succinate	1.19
3-Aminoisobutyrate	1.17	Methylamine	1.18
Valine	1.17	**Citrate**	**1.16**
Creatine	1.16	Tartrate	1.14
Acetone	1.15	3-Aminoisobutyrate	1.14
Gluconate	1.13	3-Hydroxyisovalerate	1.13
3-Hydroxyisovalerate	1.13	Valine	1.13
Tyrosine	1.12	Gluconate	1.12
Tartrate	1.09	Tyrosine	1.11
Methylamine	1.08	Dimethylsulfone	1.10
Dimethylsulfone	1.05	Methanol	1.09
Glycolate	1.05	Formate	1.08
Methanol	1.04	Glycolate	1.08
Urea	1.04	Dimethylamine	1.04
Hypoxanthine	1.03	1-Methylnicotinamide	1.02
Pseudouridine	1.03	Pseudouridine	1.02
Dimethylamine	1.02	Urea	1.00
Uracil	0.99	Creatinine	0.99
Trimethylamine N-oxide	0.98	Hypoxanthine	0.96
Creatinine	0.97	Trimethylamine N-oxide	0.96
1-Methylnicotinamide	0.94	Uracil	0.95
3-Methylxanthine	0.90	3-Methylxanthine	0.90
3-Indoxylsulfate	0.90	3-Indoxylsulfate	0.84
Trigonelline	0.74	Trigonelline	0.73
Hippurate	0.69	Hippurate	0.71

Metabolites are sorted from high to low median FCs. Bold metabolites indicate significant different fold changes between men and women
