# Supplementary material for: An NMR-Based Approach to Identify Urinary Metabolites Associated with Acute Physical Exercise and Cardiorespiratory Fitness in Healthy Humans—Results of the KarMeN Study
Source: Metabolites. 2020 May 21;10(5):212. doi: 10.3390/metabo10050212 (PMC7281079; doi:10.3390/metabo10050212)
Supplement: Supplementary file 1 [file metabolites-10-00212-s001.zip › Supplement-790661_revised/Table S4_Correlations Between VO2peak and Urinary Metabolites.pdf]

Table S4. Pearson correlation coefficients for the association between VO_{2peak} and urinary metabolite concentrations pre- and post-exercise as well as exercise-induced metabolite fold changes (based on Van der Waerden scores of the analyzed variables)

**Metabolites**	**Pre-Exercise**		**Post-Exercise**		**Fold Changes**	
	r (unadjusted)	***r (adjusted*)***	***r (unadjusted)***	***r (adjusted*)***	***r (unadjusted)***	***r (adjusted*)***
1-Methylnicotinamide	-0.10	-0.06	**-0.27**	**-0.15**	**-0.25**	**-0.13**
2-Hydroxyisobutyrate	-0.03	0.05	-0.09	0.03	-0.09	-0.04
3-Aminoisobutyrate	-0.01	0.12	-0.04	0.12	-0.12	-0.07
3-Hydroxyisovalerate	0.05	0.01	0.06	-0.05	0.00	-0.04
3-Indoxylsulfate	**-0.18**	-0.02	**-0.22**	-0.04	-0.09	-0.04
3-Methylxanthine	-0.06	0.08	-0.10	0.08	-0.08	0.04
4-Hydroxyphenylacetate	**-0.17**	0.06	**-0.16**	-0.03	-0.01	-0.05
Acetate	**-0.20**	0.02	-0.12	-0.01	0.08	-0.03
Acetone	**0.14**	0.01	0.08	-0.01	-0.09	-0.02
Alanine	-0.10	-0.08	-0.07	-0.09	0.05	-0.01
Betaine	**-0.21**	-0.06	**-0.20**	-0.03	0.01	0.04
Carnitine	0.07	-0.04	**0.15**	-0.02	0.08	0.04
cis-Aconitate	**-0.15**	-0.04	**-0.29**	-0.10	-0.13	-0.05
Citrate	**-0.46**	0.03	**-0.39**	0.06	**0.26**	0.02
Creatine	**-0.24**	-0.10	**-0.35**	-0.11	**-0.21**	-0.02
Creatinine	**0.13**	-0.02	0.02	-0.09	-0.14	-0.06
Dimethylamine	-0.08	-0.04	**-0.25**	-0.10	**-0.18**	-0.04
Dimethylsulfone	**-0.23**	0.05	**-0.22**	0.09	0.02	0.01
Formate	-0.08	0.09	0.02	0.07	**0.16**	0.01
Gluconate	**-0.30**	0.05	**-0.36**	0.04	-0.06	0.02
Glycine	**-0.23**	-0.03	**-0.17**	-0.03	0.12	0.00
Glycolate	0.06	-0.10	-0.01	-0.12	-0.09	0.00
Guanidoacetate	**-0.39**	-0.10	**-0.41**	**-0.12**	0.02	-0.01
Hippurate	**-0.30**	-0.03	**-0.34**	-0.05	-0.14	-0.05
Histidine	**0.17**	**-0.17**	**0.20**	-0.11	0.00	0.06
Hypoxanthine	-0.04	0.07	-0.11	-0.09	-0.06	**-0.13**
Isoleucine	-0.12	-0.10	**-0.14**	-0.05	-0.02	0.05
Lactate	**-0.38**	0.04	-0.09	0.04	0.09	0.04
Leucine	0.08	-0.02	0.08	-0.05	0.00	-0.04
Mannitol	0.03	0.03	-0.09	0.03	-0.10	-0.01
Methanol	0.01	0.08	0.04	0.09	0.04	0.00
Methylamine	-0.10	-0.08	**-0.19**	-0.12	-0.11	-0.02
Methylsuccinate	0.02	-0.06	-0.02	-0.10	-0.04	-0.05
N,N-Dimethylglycine	-0.01	0.03	-0.01	0.04	0.00	0.02
Pseudouridine	-0.10	0.00	**-0.22**	-0.05	-0.12	-0.04
Pyruvate	**-0.16**	-0.06	**-0.12**	-0.11	-0.03	-0.05
Succinate	**-0.32**	-0.02	**-0.31**	0.02	0.06	0.05
Tartrate	**-0.13**	0.02	**-0.20**	-0.08	-0.03	-0.05
Taurine	**0.15**	-0.12	0.10	-0.09	-0.11	0.04
Threonine	-0.01	-0.10	0.10	-0.04	**0.14**	0.03
trans-Aconitate	0.09	0.11	**-0.20**	0.07	**-0.23**	-0.02
Trigonelline	**-0.35**	0.04	**-0.36**	0.05	-0.06	-0.01
Trimethylamine N-oxide	-0.03	-0.03	-0.06	-0.07	-0.05	-0.04
Tyrosine	-0.04	**-0.16**	-0.06	**-0.18**	-0.02	-0.04
Uracil	0.06	**0.16**	0.06	0.07	0.00	-0.10
Urea	**0.15**	0.10	**0.23**	0.10	0.08	-0.01
Valine	0.02	-0.05	-0.01	-0.07	-0.03	-0.02

* correlations were adjusted for sex, menopausal status, age (y) and lean body mass (kg); bold: 95% confidence intervals do not include zero
